# Supplementary material for: Initial agronomic benefits of enhanced weathering using basalt: A study of spring oat in a temperate climate
Source: PLoS One. 2024 Mar 27;19(3):e0295031. doi: 10.1371/journal.pone.0295031 (PMC10971544; doi:10.1371/journal.pone.0295031)

S1 Figure. Average monthly temperature and monthly total precipitation from a local weather station. Shaded grey area is the range (minimum to maximum) between 2002-2021, with grey lines for the monthly mean. Superimposed (black dots and line) show the data from 2022. Data is missing from 2007, 2014 and October - December 2022 due to power shortages and/or unavailability of monitoring equipment during these periods. Monthly weather data, including months covering the growing season presented in this study (April - September), show that 2022 was an average year in terms of air temperature when compared to the long-term record. However, Rainfall was relatively low during the current study in comparison to long term trends, with total monthly precipitation in four out of the six growing season months among the lowest on record.

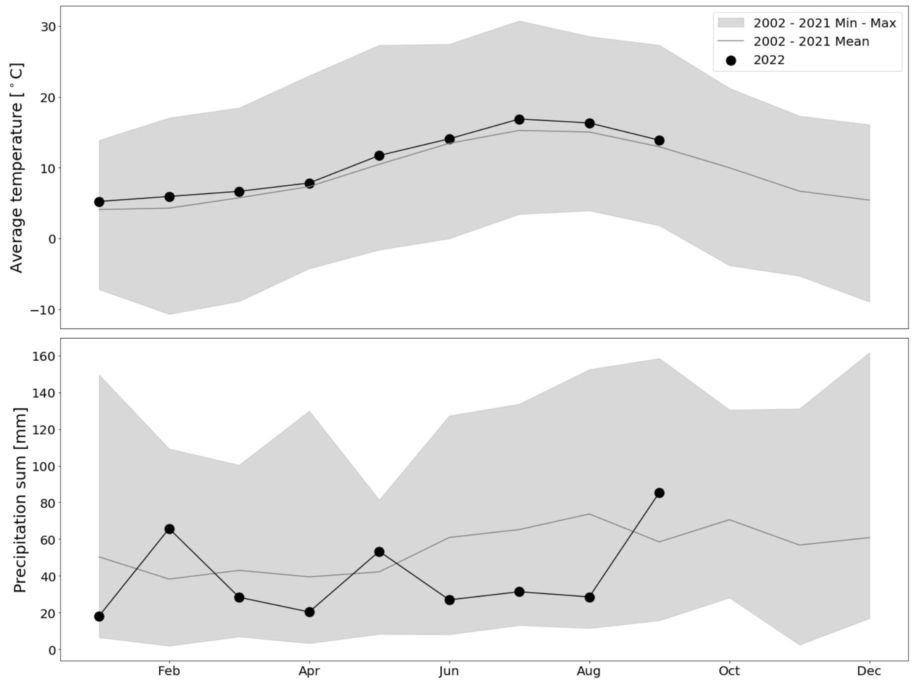

Supplement: S1 Fig — (PDF) [file pone.0295031.s001.pdf]
