# Supplementary material for: Initial agronomic benefits of enhanced weathering using basalt: A study of spring oat in a temperate climate
Source: PLoS One. 2024 Mar 27;19(3):e0295031. doi: 10.1371/journal.pone.0295031 (PMC10971544; doi:10.1371/journal.pone.0295031)
Supplement: S1 Appendix — (PDF) [file pone.0295031.s002.pdf]

S1 Appendix: Herbicide and fungicide applications

The main herbicide used was glyphosate (Apr 6th 2022, 2 L ha^{-1} AmegaDuo; 1 L ha^{-1} Firebrand (adjuvant)), with a follow up treatment on May 23rd 2022 (Paramount Max 20 g ha^{-1}, Duplosan KV 0.96 L ha^{-1}, Moraine 0.4 L ha^{-1}). Fungicides were applied on May 24th 2022 (Medax Max 0.36 kg ha^{-1}; Aurelia 0.4 L ha^{-1}; Vivid 200 0.325 L ha^{-1}; Justice 0.146 L ha^{-1}; Manganese 3 L ha^{-1}) and Jun 15th 2022 (Elatus Era 0.4 L ha^{-1}; Folmagnesium 5 L ha^{-1}).
