# Supplementary material for: Initial agronomic benefits of enhanced weathering using basalt: A study of spring oat in a temperate climate
Source: PLoS One. 2024 Mar 27;19(3):e0295031. doi: 10.1371/journal.pone.0295031 (PMC10971544; doi:10.1371/journal.pone.0295031)
Supplement: S2 Appendix — (PDF) [file pone.0295031.s003.pdf]

S2 Appendix: Basalt Characterisation

Basalt source

A 3 kg sample of crushed rock fines (0-4 mm fraction, as defined by the quarry) was taken from the Divet Hill Quarry production line (55.1003°N, -2.03459°W; Northumberland, UK), on Dec 16th, 2021. The basalt here belongs to Great Whin Sill and is identified as a Quartz-microgabbro of Carboniferous-Permian age by the British Geological Survey [1].
Mass fraction sieving of the basalt (conducted by Meritics, Leighton Buzzard, UK) showed that the crushed rock fines were taken to have a D10, D50 and D90 (i.e., the diameter at which 10%, 50% and 90% of rock fall below) of 223.7 µm, 1147 µm and 2201 µm, respectively.

Sample preparation

The samples used for Inductively Coupled Plasma - Atomic Emission Spectroscopy /- Mass spectrometry (ICP-AES and ICP-MS) were prepared by ALS (Loughrea, Ireland). To create representative samples, the sample was dried at 90°C and then crushed until greater than 70% of the sample was less than 2 mm. A 250 g rifle split of this dried and crushed material was then pulverised using a LM2 pulverising mill with B2000 steel bowls, and sieved until greater than 80% of the material was less than 75 µm.

Oxide composition

The major oxide composition of the basalt was measured using X-Ray fluorescence (XRF) at ALS. A 0.66 g sample was prepared into a fused disk. The samples were then run using an XRF. Loss on ignition (LOI) was also determined by ALS by heating the sample to 1000°C in a furnace. The XRF and LOI results can be found in (Table 2).

Elemental Composition

The ICP-AES and ICP-MS analyses were also conducted by ALS. The rock sample was first digested to a near-total digestion using a four acid digest. For this, 0.25 g of the sample was digested with perchloric, nitric and hydrofluoric acids at 185°C before the residue of the sample was finally dissolved with hydrochloric acid and then subsequently diluted before measurement. The solution produced was then analysed using an Agilent ICP-OES 5110 for all elements in Table 3 with the exception of Hg. The analytical ranges of these elements vary among the measured elements, given in Table 3.
Hg was also determined (Table 3). Here, a 0.5 g prepared sample was digested with an aqua-regia digestion at 115°C. The resultant solution was then diluted with demineralised water, mixed well and analysed using an Agilent ICP-MS 7900, with a calibrated analytical range of 0.005 - 100 ppm. Before running

samples, the instruments were calibrated using seven calibration solutions, three IEC solutions and two quality control solutions to ensure the data was robust. The analytical instrument was also calibrated by using Agilent wavelength solution. Before running samples, the instruments were calibrated using seven calibration solutions, three internal element correction solutions and two quality control solutions to ensure the data was robust. The analytical instrument was also calibrated using an Agilent wavelength solution.

Basalt mineralogy

A representative sample of Divet Hill basalt produced by ALS (dried at 70°C) was sent to X-ray Mineral Services Laboratory (Colwyn Bay, UK) for X-ray diffraction (XRD) analysis, to determine the whole rock mineralogy. The sample that was sent for analysis was then coned and quartered to produce a representative sample with a weight of 20 g. This 20 g sample was disaggregated gently using a pestle and mortar to homogenise the sample. The sample was then spiked with 10 g of $<$44 µm silicon powder at 99% purity (trace metals basis) (Sigma Aldrich, Germany) as an internal standard to allow for the determination of the amorphous phase (interpreted in this case as basaltic glass). Then, 2 g of the sample was micronised using a McCrone Micronising Mill, using zirconium elements to obtain a powder with individual particles between 5 and 10 µm in diameter. The resultant slurry was dried overnight in an oven at 80°C, then recrushed to a fine powder. The sample was 'backpacked' into an aluminium cavity mount to produce a randomly oriented sample for whole rock analysis.
The XRD powder analysis was conducted with a Malvern Panalytical X'Pert^3 diffractometer from 4.5 to 75°2θ using a CuKα radiation at 40 kV and 40 mA. The samples were analysed for 20 min with a step size of 0.013°. Rietveld analysis was then used to quantify the mineralogy using the AUTOQUAN software, with crystallographic information files taken from the ICDD PDF-4+ database. The mineralogy of the basalt determined using powder X-ray diffraction is given in Table 1.

References

[1] British Geological Survey. Geology Viewer; 2023. Available from: https://geologyviewer.bgs.ac.uk/. [Accessed 02 March 2023]
