# Supplementary material for: Initial agronomic benefits of enhanced weathering using basalt: A study of spring oat in a temperate climate
Source: PLoS One. 2024 Mar 27;19(3):e0295031. doi: 10.1371/journal.pone.0295031 (PMC10971544; doi:10.1371/journal.pone.0295031)
Supplement: Attachment — Submitted filename: PONE-D-23-15404_reviewer.pdf [file pone.0295031.s004.pdf]

PLOS ONE

Initial agronomic benefits of enhanced weathering using basalt: A study of spring oat in a temperate climate

--Manuscript Draft--

Manuscript Number:	PONE-D-23-15404
Article Type:	Research Article
Full Title:	Initial agronomic benefits of enhanced weathering using basalt: A study of spring oat in a temperate climate
Short Title:	Agronomic benefits of enhanced weathering using basalt
Corresponding Author:	Kirstine Skov<br>UNDO Carbon ltd.<br>London, UNITED KINGDOM
Keywords:	Agronomic Benefits; Enhanced Weathering; Crushed Basalt Rock; Increased Yield, CDR
Abstract:	Addressing soil nutrient degradation and global warming requires novel solutions. Enhanced weathering using crushed basalt rock may offer a partial solution, through a combined approach of sequestering carbon dioxide and improving agronomic conditions. This study examines the short-term effects of basalt amendment on spring oat (Avena sativa L.) during the 2022 growing season in NE England. The experimental design<br>consisted of four blocks with control and basalt-amended plots, and two cultivation types within each treatment, laid out in a split plot design. Basalt (18.86 tonnes ha−1) was incorporated during seeding. Tissue, grain and soil samples were collected for yield, nutrient, and pH analysis. Basalt amendment led to significantly higher yields, averaging 20.5% and 9.3% increases in direct drill and ploughed plots, respectively. Soil pH was significantly higher 256 days after application across cultivation types (direct drill: on average 6.47 vs. 6.76 and ploughed: on average 6.69 vs. 6.89, for control and basalt amended plots, respectively), likely due to rapidly dissolving minerals. Indications of growing season differences in soil pH are observed through reduced manganese and iron uptake in amended crops. Higher grain and tissue potassium, and tissue calcium uptake were observed in basalt-amended crops. In contrast, no accumulation of potentially toxic elements was detected in the grain, indicating that crops grown using this crushed basalt rock are safe for consumption. This study suggests that agronomic benefits following crushed basalt rock applications are attainable in regions with temperate climate and similar soil conditions. These findings offer valuable insights for producers in temperate climates who are considering using such amendments, demonstrating the potential for improved crop yields and environmental benefits while ensuring crop safety.
Order of Authors:	Kirstine Skov
	Jez Wardman
	Matthew Healey
	Amy Lewis
	Tzara Bierowiec
	Julia Cooper
	Ifeoma Edeh
	Dave George
	Mike Kelland
	Jim Mann
	David Manning
	Melissa J. Murphy

Powered by Editorial Manager® and ProduXion Manager® from Aries Systems Corporation

	Ryan Pape
	Yit A. Teh
	Will Turner
	Peter Wade
	Xinran Liu
**Additional Information:**	
**Question**	**Response**
**Financial Disclosure**<br><br>Enter a financial disclosure statement that describes the sources of funding for the work included in this submission. Review the submission guidelines for detailed requirements. View published research articles from *PLOS ONE* for specific examples.<br><br>This statement is required for submission and **will appear in the published article** if the submission is accepted. Please make sure it is accurate.<br><br>**Unfunded studies**<br>Enter: *The author(s) received no specific funding for this work.*<br><br>**Funded studies**<br>Enter a statement with the following details:<br>• Initials of the authors who received each award<br>• Grant numbers awarded to each author<br>• The full name of each funder<br>• URL of each funder website<br>• Did the sponsors or funders play any role in the study design, data collection and analysis, decision to publish, or preparation of the manuscript?<br>• **NO** - Include this sentence at the end of your statement: *The funders had no role in study design, data collection and analysis, decision to publish, or preparation of the manuscript.*<br>• **YES** - Specify the role(s) played.<br><br>* typeset	The study design and yield data collection was conducted by Newcastle University, independent of funders of the project.<br>All other data collection and analysis was funded by UNDO Carbon ltd.<br>Decision to publish and preparation of the manuscript was done in collaboration between Newcastle University and UNDO Carbon ltd.
**Competing Interests**<br><br>Use the instructions below to enter a competing interest statement for this submission. On behalf of all authors,	Kirstine Skov, Jez Wardman, Matthew Healey, Amy Lewis, Tzara Bierowiec, Ifeoma Edeh, Melissa J. Murphy, Ryan Pape, Will Turner, Peter Wade and Xinran Liu are presently or were recently employed in the Science and Research department and may hold options in UNDO Carbon ltd.<br>Mike Kelland is an independent consultant for UNDO Carbon ltd.

Powered by Editorial Manager® and ProduXion Manager® from Aries Systems Corporation

disclose any competing interests that could be perceived to bias this work—acknowledging all financial support and any other relevant financial or non-financial competing interests.<br><br>This statement is required for submission and **will appear in the published article** if the submission is accepted. Please make sure it is accurate and that any funding sources listed in your Funding Information later in the submission form are also declared in your Financial Disclosure statement.<br><br>View published research articles from *PLOS ONE* for specific examples.<br><br>**NO authors have competing interests**<br><br>Enter: *The authors have declared that no competing interests exist.*<br><br>**Authors with competing interests**<br><br>Enter competing interest details beginning with this statement:<br><br>*I have read the journal's policy and the authors of this manuscript have the following competing interests: [insert competing interests here]*<br><br>* typeset	Jim Mann is the founder and CEO of UNDO Carbon ltd.<br>David Manning is part of UNDO carbon ltd.s scientific advisory board.<br>Julia Cooper, Dave George and Yit A. Teh declare no conflicts of interest.
**Ethics Statement**<br><br>Enter an ethics statement for this submission. This statement is required if the study involved:<br><br>• Human participants<br>• Human specimens or tissue<br>• Vertebrate animals or cephalopods<br>• Vertebrate embryos or tissues<br>• Field research<br><br>Write "N/A" if the submission does not require an ethics statement.	N/A

General guidance is provided below. Consult the submission guidelines for detailed instructions. **Make sure that all information entered here is included in the Methods section of the manuscript.**<br><br>**Format for specific study types**<br><br>**Human Subject Research (involving human participants and/or tissue)**<br>• Give the name of the institutional review board or ethics committee that approved the study<br>• Include the approval number and/or a statement indicating approval of this research<br>• Indicate the form of consent obtained (written/oral) or the reason that consent was not obtained (e.g. the data were analyzed anonymously)<br><br>**Animal Research (involving vertebrate animals, embryos or tissues)**<br>• Provide the name of the Institutional Animal Care and Use Committee (IACUC) or other relevant ethics board that reviewed the study protocol, and indicate whether they approved this research or granted a formal waiver of ethical approval<br>• Include an approval number if one was obtained<br>• If the study involved *non-human primates*, add *additional details* about animal welfare and steps taken to ameliorate suffering<br>• If anesthesia, euthanasia, or any kind of animal sacrifice is part of the study, include briefly which substances and/or methods were applied<br><br>**Field Research**<br><br>Include the following details if this study involves the collection of plant, animal, or other materials from a natural setting:<br>• Field permit number<br>• Name of the institution or relevant body that granted permission	
**Data Availability**<br><br>Authors are required to make all data	No - some restrictions will apply

underlying the findings described fully available, without restriction, and from the time of publication. PLOS allows rare exceptions to address legal and ethical concerns. See the PLOS Data Policy and FAQ for detailed information.<br><br>A Data Availability Statement describing where the data can be found is required at submission. Your answers to this question constitute the Data Availability Statement and **will be published in the article**, if accepted.<br><br>**Important:** Stating 'data available on request from the author' is not sufficient. If your data are only available upon request, select 'No' for the first question and explain your exceptional situation in the text box.<br><br>Do the authors confirm that all data underlying the findings described in their manuscript are fully available without restriction?	
**Describe where the data may be found in full sentences. If you are copying our sample text, replace any instances of XXX with the appropriate details.**<br><br>• If the data are **held or will be held in a public repository**, include URLs, accession numbers or DOIs. If this information will only be available after acceptance, indicate this by ticking the box below. For example: *All XXX files are available from the XXX database (accession number(s) XXX, XXX.*).<br>• If the data are all contained **within the manuscript and/or Supporting Information files**, enter the following: *All relevant data are within the manuscript and its Supporting Information files.*<br>• If neither of these applies but you are able to provide **details of access elsewhere**, with or without limitations, please do so. For example:<br><br>*Data cannot be shared publicly because of [XXX]. Data are available from the*	Data available on request from the author.

Powered by Editorial Manager® and ProduXion Manager® from Aries Systems Corporation

XXX Institutional Data Access / Ethics Committee (contact via XXX) for researchers who meet the criteria for access to confidential data.<br><br>*The data underlying the results presented in the study are available from (include the name of the third party and contact information or URL).*<br>• This text is appropriate if the data are owned by a third party and authors do not have permission to share the data.<br><br>* typeset	
Additional data availability information:	Tick here if the URLs/accession numbers/DOIs will be available only after acceptance of the manuscript for publication so that we can ensure their inclusion before publication.

Powered by Editorial Manager® and ProduXion Manager® from Aries Systems Corporation

Manuscript
Click here to access/download;Manuscript;Manuscript_2023-05-19.pdf

Initial agronomic benefits of enhanced weathering using basalt: A study of spring oat in a temperate climate

Kirstine Skov[1*], Jez Wardman[1], Matthew Healey[1], Amy Lewis[1], Tzara Bierowiec[1], Julia Cooper[2], Ifeoma Edeh[1], Dave George[3], Mike Kelland[1], Jim Mann[1], David Manning[3], Melissa J. Murphy [1], Ryan Pape[1], Yit A. Teh[3], Will Turner[1], Peter Wade[1], Xinran Liu[1]

1 UNDO Carbon ltd, London, United Kingdom
2 Organic Research Centre, Cirencester, Gloucestershire, United Kingdom
3 Newcastle University, School of Natural and Environmental Sciences, Newcastle upon Tyne, Devonshire, United Kingdom

* kirstine.skov@un-do.com

Abstract

Addressing soil nutrient degradation and global warming requires novel solutions. Enhanced weathering using crushed basalt rock may offer a partial solution, through a combined approach of sequestering carbon dioxide and improving agronomic conditions. This study examines the short-term effects of basalt amendment on spring oat (*Avena sativa L.*) during the 2022 growing season in NE England. The experimental design consisted of four blocks with control and basalt-amended plots, and two cultivation types within each treatment, laid out in a split plot design. Basalt (18.86 tonnes ha^{-1}) was incorporated during seeding. Tissue, grain and soil samples were collected for yield, nutrient, and pH analysis. Basalt amendment led to significantly higher yields, averaging 20.5% and 9.3% increases in direct drill and ploughed plots, respectively. Soil pH was significantly higher 256 days after application across cultivation types (direct drill: on average 6.47 vs. 6.76 and ploughed: on average 6.69 vs. 6.89, for control and basalt amended plots, respectively), likely due to rapidly dissolving minerals. Indications of growing season differences in soil pH are observed through reduced manganese and iron uptake in amended crops. Higher grain and tissue potassium, and tissue calcium uptake were observed in basalt-amended crops. In contrast, no accumulation of potentially toxic elements was detected in the grain, indicating that crops grown using this crushed basalt rock are safe for consumption. This study suggests that agronomic benefits following crushed basalt rock applications are attainable in regions with temperate climate and similar soil conditions. These findings offer valuable insights for producers in temperate climates who are considering using such amendments, demonstrating the potential for improved crop yields and environmental benefits while ensuring crop safety.

Introduction

Nutrient limitations in agricultural soil represent a global challenge and of major concern in relation to sustaining and increasing crop yield, thereby ensuring food security to feed the growing human population [1]. Furthermore, availability of new agricultural land is limited [2] and effects of climate change are predicted to impede food production [3]. Enhanced weathering (EW) of silicate rock may aid in securing

food safety, through agronomic benefits, resulting from rock-derived nutrients [4, 5]. EW has also been proposed as a promising, scalable natural carbon dioxide removal (CDR) technology with the potential to sequester gigatons of atmospheric carbon dioxide [5, 6].

The application of silicate rocks such as basalt as EW amendments to cropland offer a long list of potential agronomic benefits [7–9]. Studies dating back to the 1960's [10, 11] and even 1930's [12, 13] have researched the benefits of applying crushed silicate rock to agricultural and forest soils. Basalt is rich in minerals that release macro- (e.g., calcium, magnesium, silicon, potassium, phosphorus, sulfur, but excluding nitrogen) and micro-nutrients (e.g., copper, iron, manganese, molybdenum and zinc) during dissolution that are essential for plant growth [4–6]. Crushed silicate rocks have been studied with respect to underestimated potassium and micronutrient deficiencies, following agricultural soil nutrient stripping [9]. Some of these micronutrients are redox-active which makes them important cofactors in enzymatic, metabolic and cellular processes [14]. Additionally, these micronutrients are actively involved in nitrogen fixation and photosynthesis [15, 16].

Increased nutrient availability may lead to increased crop yields. A synthesis study [9] found a significant yield increase in 19 out of 34 peer-reviewed papers. However, the majority (i.e. 14) of the studies which document significant yield increases were performed on highly weathered, acidic, tropical soils. Comparatively, studies in temperate climates showed less definitive evidence of yield gains, with the exception of experiments where fast weathering minerals (e.g. nepheline and biotite mica) were applied. Positive effects on yield of maize and potatoes have also been reported recently in a study using glacial rock flour in the temperate climate of southern Denmark [17].

While macro- and micro-nutrients are present within silicate rocks, they are not immediately available for plant uptake; they only become available following mineral dissolution. A slower release of nutrients may, however, be a positive trait of silicate rocks compared to conventional fertilisers, as it may reduce the likelihood of nutrient leaching or surface run-off which can subsequently contaminate groundwater and water courses [8]. Simulations using a reactive transport model and the mineralogical composition of six commercially available basaltic quarry fines suggest that some basalts have the potential to release sufficient phosphorus to substitute average P-fertiliser application to tillage crops in the UK and the United States after one year [18].

The most pronounced potential agronomic benefit of crushed silicate rock as a soil amendment is probably the stabilising effect on pH in acidic soils, as a result of alkaline products generated from mineral dissolution [4]. Increased pH in acidic soils increases plant nutrient availability by reducing the formation of insoluble nutrient compounds caused by soil acidification [4], hence potentially increasing crop yield. Previous studies have found pH increases ranging between a relatively modest 0.2 pH units up to as much as 2 pH units on soils where the starting pH was around 4.5 [9, 19, 20] following the application of crushed rock.

While silicate rocks contain many necessary elements for crop nutrition, the contribution and accumulation of trace elements that can act as potentially toxic elements (PTEs) is a concern in relation to applying EW amendments to agricultural soils [21]. Some PTEs are beneficial plant micronutrients in low concentrations, but become potentially harmful at elevated concentrations [21]. The potential accumulation of nickel and chromium are of particular concern [21]. Nickel and chromium concentrations in ultramafic minerals like olivine (approximately 1300 and 2300 mg kg^{-1}, respectively, [22]) have sparked particular concerns in relation to application on agricultural land [23, 24]. Although levels are an order of magnitude lower in basalt [25] and basalt does not necessarily contain the mineral olivine, nickel concentrations may exceed the EU soil improver limit (100 mg kg^{-1}, EU, 2019), especially if the rock is sourced from the North-Atlantic igneous province [21]. More

generally, PTEs also include arsenic, cadmium, mercury and lead [26].

An increasing number of farmers are changing field management practices from ploughing to no-till/direct drilling, in order to reduce soil erosion and maintain natural soil fertility. Both cultivation types have implications for the physical and biological conditions of the soil, and may influence the rate of basalt dissolution. Direct drilling forms the basis of no-till cultivation approaches, which involve planting the seeds directly into the soil without disturbing the soil surface [27]. Ploughing, on the other hand, is a more invasive form of tillage, which involves turning over the ground in order to loosen the soil, break up clods, and incorporate organic matter. Compared to ploughing, direct drilling may improve the soil health and thereby yield, by markedly increasing the abundance of earthworms and ability of the soil to retain organic matter and soil nutrients [27, 28]. However, depending on the soil type and condition, both direct drilling and ploughing may lead to compaction of the topsoil with reduced root penetration depth [29] and loss in yield as a consequence [30, 31].

As a land-based CDR technology, the application of EW amendments to agricultural land is projected to be highly scalable due to the abundance [32] and availability of basalt [33]. However, the availability of crushed rock alone is insufficient if landowners are not inclined to grant access to their land to apply it. It is likely that the value proposition of EW as a CDR technology to landowners will be substantially improved with demonstrated safe practice and positive effects, particularly on yield and soil pH, under climate, soil, crop and cultivation conditions that are similar to those the farmers are operating under. The aim of the current study was therefore to investigate the effect of basalt amendment on crop yield, soil pH, nutrient and PTE uptake, across two common cultivation types in an agriculturally intensive area of NE England. The study was carried out using spring oat, *Avena sativa* L., during the 2022 growing season.

Methods

Site description

The study was undertaken at the Newcastle University-managed Nafferton Farm, located in north-east England, UK (54°59'07."N, 1°53'59.4"W). The soil is predominantly classified as a uniform Dystric Stagnosol [34], with a sandy clay loam soil texture (Sand: 60.5%, Silt: 22.5% and Clay: 17.0%) and slightly acidic conditions (average pH in 2011 of 6.3) [35]. The site is located in the Köppen climate classification subtype 'marine west coast climate' [34]. Weather station data (from 2002 to 2022) was recorded on site, using an automatic weather station (S1 Fig).

Experimental design

The trial was conducted on the so-called Quality Low Input Food (QLIF) plots. The plots were originally established in 2001 and have historically been used to investigate differences in organic and conventional farming (e.g. [35]). The experimental design consists of four blocks, each containing a single replicate of four different treatments, of which two were relevant to this study; the control and basalt amended plots (Fig 1). Within the context of the QLIF platform, the control plots are managed according to standard commercial practice. Nested within each treatment are two different cultivation types, no-till/direct drilling and ploughing, see Fig 1.

Fig 1. Experimental Plot Design. The site includes four blocks (transparent grey), each 96 x 96 meters. Within each block there are two treatments: Control (green) and basalt amended plots (blue). For each treatment there are two cultivation types: no-till/direct drill (dotted) and ploughed (hashed). Each cultivation plot is 12 x 96 meters. Background: Google Hybrid. Inset: location of the Nafferton Research Farm in NE England, overlaid OpenStreetMap.

Field management

During the 2022 growing season, spring oat, was grown on the plots [36]. Basalt was applied using a conventional agricultural lime spreader on Apr 12th 2022, with an application rate of 18.86 tonnes ha^{-1}. The ploughed fields were tilled to a depth of 15-30 cm at the end of March, followed by a press within three days (using Simba Unipress). The ploughing was done prior to basalt application, hence the basalt was incorporated to the seeding depth across both cultivation types. Both control and basalt amended plots were fertilised with liquid NitroFlow Urea Ammonium Nitrate (UAN) on Apr 27th 2022, with an application rate of 246 L ha^{-1}, providing 75.5 kgN ha^{-1} and 23.6 kgS ha^{-1}. In addition to the fertiliser application, the plots were also treated with conventional applications of herbicides and fungicides (S1 Appendix).

The seeding rate across both cultivation types was 180 kg ha^{-1}, where seeds were drilled into the soil using a John Deere 750A at a target depth of 2 cm on Apr 15th 2022. For both the direct drill and ploughed plots, the seed beds were rolled within two days (using a Cambridge roller). All fields were harvested on Sep 16th 2022 using a plot combine (Deutz-Fahr M660).

Soil sampling and analysis

Soil samples were collected on Feb 1st-2nd 2023, prior to any management activities for the impending growing season. Historically, each cultivation plot had been divided into several different treatments, with four evenly-spaced subplots [37]. These subplots were abandoned in 2017, but in order to avoid any potential legacy bias in soil pH, four soil samples were collected from each subplot for the current study, resulting in a total number of 16 soil samples from each cultivation plot, 64 samples from each block and 256 samples in total. The samples were taken to a depth of 15 cm and dried for five days at 40°C. The samples were then sieved to obtain the 2 mm fraction and analysed for pH using a 1:2.5 mixing ratio of soil to H_2O. The pH was determined using a Metrohm 914 pH/conductometer.

Plant tissue and grain sampling

The plant tissue sampling was conducted on Aug 1st 2022, by collecting 100 fully expanded flag leaves from the centerline of each of the 16 plots. The crops were at growth stage 77-81 at the time of sampling, just prior to the onset of senescence. The fresh samples were packaged in plastic zip lock bags, stored at ambient temperature and sent to the lab on the same day. The samples were received at the lab on Aug 3rd 2022. The tissue samples were dried at 60°C and milled to less than 1 mm.

The prepared tissue samples were analysed for the following nutrient suite: calcium, magnesium, manganese, boron, copper, molybdenum, iron, zinc, sulfur, phosphorus and potassium, using nitric acid microwave digestion and elemental determination through inductively coupled plasma - optical emission spectrometry (ICP-OES). The nitrogen content in the tissue samples was determined using the Dumas method and LECO Trumac instrumentation.

The grain yield was determined using a plot combine (Deutz-Fahr M660), driven in a 2.1 m strip down the centerline of each plot, with grain samples taken during combining on Sep 16th 2022. In each plot four sub samples of approximately 100 g each, were manually collected from the combine at regular intervals, merged into a composite sample, air dried at 30°C and stored in zip lock bags prior to shipping for analysis on Nov 1st 2022. The samples were analysed for the same nutrient and nitrogen suite as the tissue samples (see above), as well as for silica. Furthermore, the grain samples were analysed for the PTEs; arsenic, cadmium, chromium, lead, mercury and nickel. Both PTEs and silica were determined using aqua regia extraction and microwave digestion and elemental determination through ICP-OES.

Both plant tissue and grain samples were analysed using the United Kingdom Accreditation Service (UKAS) and ISO accredited (ISO/IEC 17025:2017) Lancrop Laboratories.

Basalt

The basalt used in this study was from the Divet Hill quarry (55.1003°N, -2.03459°W; Northumberland, UK). The composition of the basalt was investigated using x-ray fluorescence (XRF) for oxide composition, ICP-AES and ICP-MS for elemental composition and x-ray diffraction (XRD) for mineralogy (S2 Appendix). The mineralogy of the basalt can be found in Table 1. This mineralogy is consistent with interpretations identified in [38], which identified products of hydrothermal alteration (quartz, calcite) and alteration of augite (to chlorite and smectite). The particle size ranges from 0-4 mm with a median particle size of 1147 µm (S2 Appendix).

Phase, identified from XRD	Mineral formula	Acid rate constant	Neutral rate constant	wt.%
Fast dissolving silicate phases				
Andesine	$Na_{0.7-0.5}Ca_{0.3-0.5}Al_{1.3-1.5}Si_{2.7-2.5}O_8$	-8.88	-11.47	38.5
Augite	$(Ca,Na)(Mg,Fe,Al,Ti)(Si,Al)_2O_6$	-6.82	-11.97	16.8
Amphibole	$(Na,Ca,Fe,Mg,Al)_7Si_8O_{22}(OH)_2$	-7.00[a]	-10.3[a]	1.2
Medium dissolving silicate phases				
Chlorite + chlorite/smectite	$(Mg,Fe)_3(Si,Al)_4O_{10}(OH)_2{\cdot}(Mg,Fe)_3(OH)_6$	-11.11[b]	-12.52[b]	7.8
Illite+mica	$(K,Na,Ca)_2(Al,Mg,Fe)_{4-6}(Si,Al)_8O_{20}(OH,F)_4$	-11.85[c]	-13.55[c]	7.3
Slow dissolving silicate phases				
Quartz	SiO_2	-13.40	-16.29	11.4
Smectite	$(Ca,Na)_{0.25-0.33}(Al,Mg,Fe)_{2-3}((Al,Si)_4O_{10})(OH)_2 \cdot nH_2O$	-10.98	-12.78	1.4
Non-silicate phases identified				
Calcite	$CaCO_3$	-0.30	-5.81	3.6
Ilmenite	$(Fe,Ti)_{2.0}O_3$	-8.35	-11.16	2.5
Un-known silicate phases				
Amorphous	-	-	-	9.6
Total	-	-	-	100.1

[a] hornblende dissolution rate used for amphibole rates provided here
[b] chlorite dissolution rate constants used for chlorite + chlorite/smectite rates provided here
[c] muscovite dissolution rate used for illite+mica rates provided here

Table 1. Mineralogy of the Divet Hill basalt as determined using X-ray diffraction. Proportions of each mineral provided is in wt.%. Where minerals form in solid solution, general mineral formulas are provided. Acid and neutral rate constants represent log(dissolution rate) at 25°C. These constants are taken from [39].
.

Statistical analysis

A linear mixed effect model (LMEM) was used to evaluate whether the effect of basalt treatment and cultivation type was statistically significant. The treatment and cultivation factors were included as fixed effect terms and the block factor as the

random effect term. Model assumptions regarding normality and homoscedasticity of variance of residuals were tested using Shapiro-Wilk and White's lagrange multiplier tests, respectively. Where model residuals do not meet LMEM assumptions, the data was Box Cox transformed to determine the significance of the fixed effects. The potential interaction effect of the fixed effects was investigated using both a LMEM and a two-way ANOVA with interaction terms, but generally no interaction effects were found (data not shown). For pH, the statistical analysis was performed on the antilog of the negative pH value, in order to be able to consider untransformed hydrogen concentration as a continuous variable [40]. All statistical analyses were carried out using Python (version 3.9) and the package Statsmodels (version 0.13.2).

Results

Grain Yield

A significantly higher yield in the basalt amended plots was observed across cultivation types (on average 20.5% and 9.3% for direct drill and ploughed plots, respectively), as well as a higher yield in the ploughed plots compared to the direct drill plots (Table 2 and 3, Fig 2). The mean thousand grain weight in each group was notably similar (ranging from 41.14 - 41.59 g), indicating that the significant differences in grain yield were not due to larger or heavier grains, but rather a larger number of grains. It is also worth noting that a higher variance was observed within the direct drill plots compared to the ploughed plots (Fig 2).

		Units	Control + Direct Drill		Basalt + Direct Drill		Control + Ploughed		Basalt + Ploughed	
			M	***SD***	***M***	***SD***	***M***	***SD***	***M***	***SD***
Soil	pH		6.47	0.30	6.76	0.37	6.69	0.29	6.89	0.25
Grain	Yield	t ha^{-1}	3.65	0.95	4.4	0.96	5.4	0.7	5.9	0.27
	TGW	g	41.51	1.67	41.59	1.64	41.81	2.05	41.14	2.37
	Nitrogen	%	2.23	0.02	2.10	0.15	2.00	0.10	1.99	0.07
	Phosphorus	%	0.40	0.04	0.41	0.04	0.37	0.03	0.38	0.04
	Potassium	%	0.42	0.04	0.44	0.04	0.39	0.03	0.42	0.03
	Sulfur	%	0.19	0.01	0.19	0.01	0.18	0.01	0.18	0.00
	Calcium	%	0.12	0.01	0.12	0.01	0.11	0.01	0.11	0.01
	Magnesium	%	0.14	0.01	0.14	0.01	0.13	0.01	0.14	0.01
	Boron	mg kg^{-1}	0.96	0.11	1.02	0.14	0.87	0.12	0.98	0.05
	Copper	mg kg^{-1}	5.88	0.22	5.45	0.39	4.95	0.31	5.10	0.24
	Iron	mg kg^{-1}	57.05	4.68	58.97	6.61	57.98	3.54	57.83	2.71
	Manganese	mg kg^{-1}	42.60	6.96	35.30	5.24	43.40	1.87	42.20	6.23
	Molybdenum	mg kg^{-1}	0.15	0.09	0.23	0.08	0.14	0.03	0.17	0.08
	Silica	mg kg^{-1}	181.50	32.44	218.00	12.19	206.50	37.60	201.25	30.50
	Zinc	mg kg^{-1}	39.48	3.85	36.68	3.51	32.22	3.04	32.30	1.61
Grain PTEs	Arsenic	mg kg^{-1}	0.08	0.08	0.07	0.06	0.08	0.08	0.16	0.08
	Cadmium	mg kg^{-1}	0.05	0.01	0.05	0.01	0.03	0.01	0.04	0.01
	Chromium	mg kg^{-1}	4.19	0.47	4.24	0.4	4.18	0.1	3.91	0.52
	Nickel	mg kg^{-1}	2.97	0.33	2.84	0.22	3.03	0.45	2.82	0.21
Tissue	Nitrogen	%	2.7	0.16	2.42	0.05	1.92	0.16	2.02	0.22
	Phosphorus	%	0.14	0.03	0.13	0.01	0.12	0.01	0.12	0.01
	Potassium	%	1.26	0.14	1.38	0.18	1.57	0.27	1.71	0.11
	Sulfur	%	0.58	0.03	0.58	0.05	0.37	0.03	0.4	0.05
	Calcium	%	1.42	0.12	1.58	0.07	1.41	0.1	1.5	0.09
	Magnesium	%	0.24	0.01	0.23	0.02	0.19	0.03	0.21	0.03
	Boron	mg kg^{-1}	11.13	1.08	11.83	0.19	9.9	1.1	10.6	0.34
	Copper	mg kg^{-1}	5.95	0.62	5.3	0.14	4.03	0.46	4.05	0.26
	Iron	mg kg^{-1}	96.25	6.85	87	6.38	88.25	9.64	81.5	4.65
	Manganese	mg kg^{-1}	21.33	4.91	15.93	3.46	26.05	11.7	15.3	3.37
	Molybdenum	mg kg^{-1}	0.51	0.09	0.66	0.19	0.63	0.21	0.54	0.2
	Zinc	mg kg^{-1}	12.5	1	11.75	0.96	9.25	0.5	9.5	1.29

Table 2. The mean (M) and standard deviation (SD) of each determined parameter within each combination of treatment and cultivation type ($n = 4$). The grain samples were also analysed for the potentially toxic elements (PTEs) mercury and lead, but levels were below detection, and hence data for these elements are not included in this table.

		Units	Cultivation	Basalt
Soil	pH		**<0.001**	**<0.001**
Grain	Yield	t ha^{-1}	**<0.001**	**0.015**
	TGW	g	*0.933*	*0.753*
	Nitrogen	%	**<0.001**	0.135
	Phosphorus	%	**0.005**	0.320
	Potassium	%	**0.008**	**0.008**
	Sulfur	%	***<0.001***	*0.670*
	Calcium	%	0.059	0.637
	Magnesium	%	0.206	0.527
	Boron	mg kg^{-1}	**0.034**	**0.007**
	Copper	mg kg^{-1}	**<0.001**	0.402
	Iron	mg kg^{-1}	0.960	0.692
	Manganese	mg kg^{-1}	0.051	**0.031**
	Molybdenum	mg kg^{-1}	0.276	0.073
	Silica	mg kg^{-1}	0.733	0.195
	Zinc	mg kg^{-1}	**<0.001**	0.300
Grain PTEs	Arsenic	mg kg^{-1}	0.195	0.335
	Cadmium	mg kg^{-1}	**0.036**	0.675
	Chromium	mg kg^{-1}	0.250	0.455
	Nickel	mg kg^{-1}	0.888	0.145
Tissue	Nitrogen	%	**<0.001**	0.330
	Phosphorus	%	**0.002**	0.255
	Potassium	%	**<0.001**	**0.014**
	Sulfur	%	**<0.001**	0.417
	Calcium	%	0.175	**<0.001**
	Magnesium	%	**<0.001**	0.639
	Boron	mg kg^{-1}	**<0.001**	**0.026**
	Copper	mg kg^{-1}	**<0.001**	0.126
	Iron	mg kg^{-1}	**0.025**	**0.008**
	Manganese	mg kg^{-1}	0.700	**<0.001**
	Molybdenum	mg kg^{-1}	0.973	0.660
	Zinc	mg kg^{-1}	**<0.001**	0.610

Table 3. p-values for each parameter, as derived from linear mixed effect modelling with cultivation and basalt amendment as fixed effects and the block factor set as a random effect. Bold numbers show a significant effect of the fixed factors at a 95% confidence level. Italicised numbers are for parameters where model residuals were not normally distributed (Shapiro-Wilk p-value <0.05), the models did converge and there was homogeneity in the variance of the residuals. Tissue manganese and phosphorus were BoxCox transformed to satisfy normality and homoscedasticity of model residuals.

Fig 2. Yield data. Yield from basalt amended plots plotted against yield from untreated plots, paired using cultivation and block number. Mean yield from both cultivation types is visualised with the standard deviation as error bars. Dotted line indicates the 1:1 line.

Soil pH

A similar pattern to grain yield was seen in the soil pH data, where a significantly higher pH was observed in the basalt amended soil, compared to the control plots, both for the direct drill (mean 6.76 and 6.47, respectively) and ploughed plots (mean 6.89 and 6.69, respectively) (Table 2). A significant difference was also found as a result of cultivation type, with a slightly higher pH in the ploughed plots (Fig 3, Table 2 and 3).

Fig 3. Soil pH data. Box plots of soil pH from basalt amended and control plots for each cultivation type (n = 64). Boxes depict the quartile of each group, whiskers show the extent of the distribution, diamond points have been classified as outliers. The pH was determined in a 1:2.5 soil:H_2O ratio.

Grain nutrients

Significantly higher grain nutrient concentrations were observed, as a result of basalt amendment for potassium and boron, whereas there is a significantly lower concentration of manganese (Table 3). Significantly lower grain concentrations from the ploughed plots was observed for nitrogen, phosphorus, potassium, sulfur, copper, boron and zinc (Table 2).

Grain potentially toxic elements

No statistically significant accumulation of PTE concentrations were found as a result of basalt amendment (Table 3). Copper and zinc were analysed as part of the standard nutrient analysis, as well as part of the PTE suite, but only reported under nutrients, as the concentrations are far below toxicity (Table 2). A slightly lower uptake of cadmium was observed as a result of ploughing ($p<0.05$, Table 2 and 3). Lead and mercury are not reported as concentrations were below the detection limit (0.5 mg kg-1).

Tissue nutrients

Significantly higher tissue concentrations of calcium, potassium and boron were found in the basalt amended plots (Table 2), whereas significantly lower concentrations of manganese and iron were found. Similar to the grain nutrient concentrations, all nutrients (nitrogen, phosphorus, sulfur, magnesium, iron, copper, boron and zinc) except potassium were significantly higher in plant tissues in the direct drill plots relative to the ploughed treatments (Table 2 and 3).

Discussion

The aim of this study was to examine any first year effects of applying crushed basalt to agricultural fields. The study focuses on yield responses of spring oat, tissue and grain nutrient and PTE uptake, as well as differences in soil pH between basalt amended and control fields.

Yield

The basalt amendment appears to show immediate, short-term benefits for crop yield, with a significantly higher yield observed across both ploughed and direct drill fields (Table 3, Fig. 2). Concurrently, the average mass of one thousand grains across all four groups did not vary, ranging between 41.14 and 41.81 g (Table 2), indicating that the higher yield was not due to larger or heavier grain, but rather a higher abundance of grain.

The difference in mean yield due to basalt amendment on the direct drill plots was 0.75 tonnes ha^{-1} (i.e. 4.40 vs 3.65 tonnes ha^{-1}) and on the ploughed plots 0.50 tonnes ha^{-1} (i.e. 5.90 vs 5.40 tonnes ha^{-1}), see Table 2. This corresponds to a mean difference of 20.5 and 9.3%, respectively, although it should be noted that the variance within each group was relatively large (with standard deviations ranging from 0.27-0.95 tonnes ha^{-1}, Table 2). First year differences in yield were also observed for potato and maize in a study in southern Denmark [17], although the median particle size of the glacial rock flour used in their study is considerably smaller than the size in this study (2.6 µm vs. 1147 µm). A similar average increase of 21% in *Sorghum bicolor* yield was found in a controlled mesocosm experiment with an application rate of 100 tonnes ha^{-1} (a fivefold increase in basalt application rate compared to this study), sufficient irrigation and controlled temperatures [41]. An even greater increase of 100% in soybean yield was reported in a Canadian microplot experiment [42], with the magnitude of this increase potentially explained by the use of the fast-dissolving mineral wollastonite, again delivered at a relatively high application rate of 100 tonnes ha^{-1}.

The findings in this study are noteworthy because the experimental boundary conditions were expected to lead to lower rates of dissolution compared to the studies discussed above and hence smaller crop yield responses. The current study was confined by a basalt particle size up to 4 mm, a reduced application rate, a relatively dry growing season (S1 Fig) and minimal basalt incorporation into the soil. Hence, positive effects of the basalt amendment were not expected during the first growing season. However, given the dry growing season conditions, the crop grown in the basalt amended plots may have benefited from the silicon released from fast dissolving silicate minerals, used by plants to strengthen plant tissue walls and making the crop more drought resistant subsequently leading to higher yields [43–45]. Whether the significantly higher yield due to basalt amendment would have occurred in a growing season that was not relatively dry will need to be studied in future growing seasons. However, it may be an indication that soil amendments using crushed basalt have the potential to make crops more resilient to drier conditions as a result of climate change.

Statistically, the strongest effect on crop yield was that of cultivation type ($p<0.005$, Table 3), with a difference in the mean yield across basalt amendment of approximately 2 tonnes ha^{-1} (Fig. 2). However, the variance within the direct drill groups, for both the control and basalt amended treatments, was much higher than in the ploughed groups (Table 2). It could be speculated that poor establishment in the direct drill plots gave rise to this result, with an uneven and patchy crop stand observed throughout the growing season. The direct drill cultivation was introduced in 2019 and drilling dates and seeding rates kept constant with the ploughed plots since then. These factors could have been expected to result in poorer performance, particularly on heavy clay soils in

temperate climates where yield depression after transitioning to direct drilling methods is common (especially in the initial years after switching from ploughing [46]), and often commercially compensated for by varying drilling dates and increasing seeding rates. This may also explain why the findings here contradict those of other studies, where substantial increases in crop yield and aboveground biomass have been obtained from direct drill fields [47]. Soil moisture conditions at the time of drilling may have also been unfavourable in the direct drill plots, leading to the high variability seen within these.

No interaction effect was found between the cultivation types and basalt amendment for yield, this being similar to all other parameters modelled (data not shown). The lack of any interaction effect may be due to the fact that the basalt was incorporated during seeding only, with a target depth of 2 cm, for both ploughed and direct drill plots; i.e. the basalt was applied after tillage and not incorporated mechanically into the soil on the ploughed plots. Hence, in this study, the two cultivation types are not representing different basalt incorporation strategies, but rather differences in the underlying soil structure, for which the effect of the basalt amendment appears agnostic.

Soil pH

Basalt amendment led to a significantly higher soil pH relative to the control plot, in both the direct drill (on average 6.47 vs 6.76) and the ploughed plots (on average 6.69 vs. 6.89)(Table 2). Hence, a moderate, but significantly higher pH (on average 0.29 and 0.2 pH units) was observed 256 days after basalt application. Although soil samples were not collected during the growing season, it can be surmised that the significant difference in yield seen with basalt application was probably driven by modest differences in soil pH that manifested during the crop cycle. In support, indications of differences in pH between the plots during the growing season were observed in the significantly reduced uptake of manganese and iron in the basalt amended plots (Table 2 and 3). The plant availability of these nutrients decreases abruptly above pH 6.5 [4, 48, 49]. Although care should be taken when assessing the absolute pH levels, the pH of the soil in this study varied around pH 6.5 (6-7.5, Fig.3).

The mean difference in soil pH seen here is larger than that observed when using the fast dissolving mineral, wollastonite, applied at 30 tonnes ha^{-1} (no reported increase from a starting pH of 6.63 over 98 days, [42]), despite the dry growing season conditions that prevailed in this study. Nevertheless, [50] reported a change of 0.5 pH units (from 4.7 to 5.2) within 9 months of application of 20 tonnes ha^{-1} basalt. Dissolution rates would be expected to be more favourable in the study by [50], where the particle size used was less than 250 µm, and the study was conducted in a Malaysian cocoa plantation with tropical temperatures and precipitation.

Compared to the above studies, boundary conditions in this study are less favourable, and so the difference in soil pH between the control and basalt amended plots could have been anticipated to be smaller within the first year after application. The significant first year differences in pH may be ascribed to the 3.6 wt.% content of fast dissolving calcite (Table 1). The calcite (calcium carbonate) fraction contribution in this study was 0.68 tonnes ha^{-1}. Comparatively, if a limestone product with a 70% effective calcium carbonate equivalent and an application density of 2 to 5 tonnes ha^{-1} (similar to a limestone product used in [51] had been used, the contribution would range between 1.4 to 3.5 tonnes ha^{-1} calcium carbonate (between two and fivefold higher concentrations than that of the basalt). A study of six basalts from widely different geological provinces found that basalts contain up to 1.25 wt.% carbonates [52], compared to which the content in Divet Hill is relatively high. The fast dissolving silicate minerals andesine, augite, amphibole and possibly the amorphous phase will probably also have contributed to the difference in pH, although the neutral dissolution rates (which would be dominant under the pH conditions that the soil at the study site

is exposed to) are at least 30,000 times lower than the dissolution rate for calcite (Table 1). Any calcite contribution (assuming it's in the available fraction of the crushed basalt rock), may help elevate soil pH in the short term, whereas the dissolution of silicate minerals will have a longer term stabilising effect on the soil pH, due to their slower dissolution rates (Table 1). Hence, basalts containing calcite may be attractive for producers looking to immediately remediate the pH of their soil. It is not possible to determine the relative contribution of calcite and silicate mineral dissolution to the difference in pH with the present dataset.

A significantly lower pH in the direct drill plots relative to the ploughed plots was also observed (Table 3). This difference may be due to increased conservation of soil organic matter in the direct drill plots since implementation of this cultivation type in 2019 [53], although this was not investigated in the current study.

Nutrient uptake

First year differences in nutrient uptake include a significantly higher concentration of potassium in both grain and tissue and a higher concentration calcium in the plant tissue, in samples taken from the basalt amended plots. The significantly higher grain potassium concentration supports results obtained by [41], whereas the higher tissue concentration of potassium was not observed by [41]. The significantly higher concentration of potassium, both in tissue and grain, across cultivation types is not likely to be an indication of changes to soil pH, as the availability of potassium is not generally assumed to increase with changes in pH within the ranges observed in this study [49, 54]. It is therefore hypothesised that the elevated potassium observed in tissue and grain will have originated from the dissolution of potassium-bearing basaltic phases.

Potassium release may have originated from the illite/mica phase identified through XRD, as potassium is a key component of illite and several mica minerals (e.g. muscovite and biotite). Although this phase is generally considered to have a medium dissolution rate, similar to calcium and magnesium, potassium may be preferentially released into the soil solution before the silicon-oxygen bonds are broken, as hypothesised [55] and observed [56] in work elsewhere. Given the biotic nature of soils, the potassium may have also been preferentially mined from the mineral structure by microorganisms, including mycorrhizal fungi [57], though this hypothesis was not investigated in this study.

A significantly higher calcium uptake in plant tissue from the basalt amended plots was observed across cultivation types, whereas no such difference was observed for magnesium. Both calcium and magnesium are considered among the most easily weathered base cations in basalt, and are thought to be preferentially leached from minerals before the breakdown of silicon-oxygen bonds within minerals [55]. The Divet Hill basalt contains calcium (CaO: 8.97 wt.%) and magnesium (MgO: 5.32 wt.%) (S1 Table), and both cations are present in the fast dissolving silicate phases (Table 1). However, calcium is also present in the faster dissolving calcite (Table 1), which is thought to be the main source of calcium during the first growing season. In a tropical mesocosm study, using a basalt with similar chemical composition as Divet Hill (CaO: 8.92 wt.% and MgO: 5.36 wt.%) and a sandy clay loam soil, a significant increase in both calcium and magnesium content of plant shoots was observed in maize and soy [51], suggesting that magnesium may also be provided through basalt application. Although this was not evidenced during the first growing season in the current study, it may be manifested during following growing seasons. However, the absence of elevated magnesium plant uptake is not necessarily due to the absence of basalt dissolution. [41] found no magnesium increase in plant roots and shoots, but showed a significant decrease in magnesium in basalt grains extracted from the experiment (compared to unweathered basalt grains). Further, in [41] there was a significant increase in

magnesium on the soil exchangers (as interpreted from ammonium acetate-extractions) which suggests that some elements might not be as available to plants, compared to other elements.

Boron levels, in both grain and plant tissue, were also significantly higher in response to basalt amendment of soils in the current study. Reasons for this remain unclear and warrant further study, though it could be speculated that boron in anionic form is taken up in association with potassium and calcium cations.

Wherever significant differences were seen in grain and tissue analysis between the two cultivation types (Table 3), the nutrient concentrations in the direct drill plots were always higher, with the sole exception of tissue potassium (Table 2). Although direct drill systems may provide advantages for retaining soil nutrients, this elevated uptake is probably related to the poor crop stand in these plots, resulting in reduced competition for nutrients with neighbouring plants.

Potentially toxic elements

There was no evidence that application of crushed basalt rock led to accumulation of any PTEs in grain, suggesting that crops amended with this product are safe for human and animal consumption (Table 2 and 3). Product toxicity was not unexpected in the current study, particularly given that element concentrations in the Divet Hill basalt do not exceed any of the EU thresholds for inorganic soil improver products (S2 Table). Dietary limits for PTEs in cereals have been found for cadmium (0.1 mg kg^{-1}) and lead (0.2 mg kg^{-1}), mercury in fishery products (0.5 mg kg^{-1}, [58]) and for arsenic in wheat (1 mg kg^{-1}, [59]). The grain concentrations in this study were well below these limits, with lead and mercury being undetectable and maximum concentrations for cadmium and arsenic of 0.07 mg kg^{-1} and 0.26 mg kg^{-1}, respectively. Although no dietary limits for chromium and nickel were found, it's reassuring that no significant differences in grain concentrations were observed for these elements (Table 3).

Conclusion

The application of crushed basalt rock resulted in significantly higher spring oat yields across direct drill and ploughed plots, during the first growing season. The difference in yield between the control and basalt amended plots was primarily ascribed to a modest difference in soil pH, which during the growing season materialised in reduced grain and tissue manganese uptake, as well as reduced tissue iron uptake. The reported differences in pH were most likely primarily a result from the fast dissolution of calcite, as well as silicate mineral dissolution. The former is supported by significantly higher tissue calcium content, whereas the latter is supported by a significantly higher grain and tissue potassium, in the crops from the basalt amended plots. The significant differences that arose due to basalt amendment were agnostic to cultivation type, and hence the underlying soil structure, probably because of the lack of mechanical incorporation of basalt into the ploughed soil. Differences in yield and plant nutrients due to cultivation were largely ascribed to poor emergence (and thus lower competition for nutrients between plants) of spring oat grown in the direct drill plots. Basalt application did not result in the accumulation of PTEs in grains compared to the control plots, indicating that crops amended with crushed basalt rock are safe for consumption.

Supporting information

S1 Fig Weather data

S1 Appendix Herbicide and fungicide applications

S2 Appendix Basalt characterisation

S1 Table Basalt oxide composition

S2 Table Basalt elemental composition

Acknowledgments

The authors would like to thank all the employees at the Nafferton research farm that were involved in this work, including (but not limited to) James Standen, Gavin Hall and Rachel Chapman.

References

1. Manning DAC, Theodoro SH. Enabling food security through use of local rocks and minerals. Extr Ind Soc. 2020;7:480–487. doi:10.1016/j.exis.2018.11.002.

2. Hossain A, Krupnik TJ, Timsina J, Mahboob MG, Chaki AK, Farooq M, et al. 2. In: Agricultural Land Degradation: Processes and Problems Undermining Future Food Security. Cham, Switzerland: Springer Nature Switzerland AG; 2020. p. 17–61.

3. Lal R. Food security in a changing climate. Ecohydrol & Hydrobiol. 2013;13:8–21. doi:10.1016/j.ecohyd.2013.03.006.

4. Kantola IB, Masters MD, Beerling DJ, Long SP, DeLucia EH. Potential of global croplands and bioenergy crops for climate change mitigation through deployment for enhanced weathering. Bio Lett. 2017;13:20160714. doi:10.1098/rsbl.2016.0714.

5. Beerling DJ, Kantzas EP, Lomas MR, Wade P, Eufrasio RM, Renforth P, et al. Potential for large-scale CO2 removal via enhanced rock weathering with croplands. Nature. 2020;583:242–248. doi:10.1038/s41586-020-2448-9.

6. Hartmann J, West AJ, Renforth P, Köhler P, Rocha CLDL, Wolf-Gladrow DA, et al. Enhanced chemical weathering as a geoengineering strategy to reduce atmospheric carbon dioxide, supply nutrients, and mitigate ocean acidification. Rev Geophys. 2013;51:113–149. doi:10.1002/rog.20004.

7. Jones DL, Cross P, Withers PJA, DeLuca TH, Robinson DA, Quilliam RS, et al. REVIEW: Nutrient stripping: the global disparity between food security and soil nutrient stocks. J Appl Ecol. 2013;50:851–862. doi:10.1111/1365-2664.12089.

8. Ramos CG, Hower JC, Blanco E, Oliveira MLS, Theodoro SH. Possibilities of using silicate rock powder: An overview. Geosci Front. 2022;13:101185. doi:10.1016/j.gsf.2021.101185.

9. Swoboda P, Döring TF, Hamer M. Remineralizing soils? The agricultural usage of silicate rock powders: A review. Sci Total Environ. 2022;807:150976. doi:10.1016/j.scitotenv.2021.150976.

10. Villiers ODD. Soil rejuvenation with crushed basalt in Mauritius Part I - Consistent results of worldwide interest. Int Sugar J. 1961;63:363–364.

11. Villiers ODD. Soil rejuvenation with crushed basalt in Mauritius Part I-The fertility of basalt and its nutritional effects. Int Sugar J. 1961;63:3–5.

12. Albert R. On the use of fine debris from stone quarries for the improvement of poor forest soils. Forstarchiv. 1938;14:229–240.

13. Hilf HH. The manuring of poor soils with basalt grit. Forstarchiv. 1938;14:93–100.

14. Hänsch R, Mendel RR. Physiological functions of mineral micronutrients (Cu, Zn, Mn, Fe, Ni, Mo, B, Cl). Curr Opin Plant Biol. 2009;12:259–266. doi:10.1016/j.pbi.2009.05.006.

15. Islam M, Mohsan S, Ali S. Interactive Effect of Sulfur and Phosphorus on Nodulation, Nitrogen Fixation, and Nutrient Uptake by Chickpea Grown on Calcareous Soils in Pakistan. J Plant Nutr. 2013;36:1649–1658. doi:10.1080/01904167.2013.805220.

16. Kumar S, Kumar S, Mohapatra T. Interaction Between Macro- and Micro-Nutrients in Plants. Front Plant Sci. 2021;12. doi:10.3389/fpls.2021.665583.

17. Gunnarsen KC, Jensen LS, Rosing MT, Dietzen C. Greenlandic glacial rock flour improves crop yield in organic agricultural production. Nutr Cycl Agroecosyst. 2023;126:51–66. doi:10.1007/s10705-023-10274-0.

18. Lewis AL, Sarkar B, Wade P, Kemp SJ, Hodson ME, Taylor LL, et al. Effects of mineralogy, chemistry and physical properties of basalts on carbon capture potential and plant-nutrient element release via enhanced weathering. Appl Geochem. 2021;132:105023. doi:10.1016/j.apgeochem.2021.105023.

19. Silva B, Paradelo R, Vázquez N, García-Rodeja E, Barral MT. Effect of the addition of granitic powder to an acidic soil from Galicia (NW Spain) in comparison with lime. Environ Earth Sci. 2013;68:429–437. doi:10.1007/s12665-012-1747-3.

20. de Lima Dias KG, Guimarães PTG, do Carmo DL, Reis THP, de Jesus Lacerda JJ. Alternative sources of potassium in coffee plants for better soil fertility, productivity, and beverage quality. Pesqui Agropecu Bras. 2018;53:1355–1362. doi:10.1590/s0100-204x2018001200008.

21. Dupla X, Möller B, Baveye PC, Grand S. Potential accumulation of toxic trace elements in soils during enhanced rock weathering. Eur J Soil Sci. 2023;74. doi:10.1111/ejss.13343.

22. Vink JPM, Knops P. Size-Fractionated Weathering of Olivine, Its CO2-Sequestration Rate, and Ecotoxicological Risk Assessment of Nickel Release. Minerals. 2023;13:235. doi:10.3390/min13020235.

23. ten Berge HFM, van der Meer HG, Steenhuizen JW, Goedhart PW, Knops P, Verhagen J. Olivine Weathering in Soil, and Its Effects on Growth and Nutrient Uptake in Ryegrass (Lolium perenne L.): A Pot Experiment. PLoS One. 2012;7:e42098. doi:10.1371/journal.pone.0042098.

24. Amann T, Hartmann J, Struyf E, de Oliveira Garcia W, Fischer EK, Janssens I, et al. Enhanced Weathering and related element fluxes – a cropland mesocosm approach. Biogeosciences. 2020;17:103–119. doi:10.5194/bg-17-103-2020.

25. Beerling DJ, Leake JR, Long SP, Scholes JD, Ton J, Nelson PN, et al. Farming with crops and rocks to address global climate, food and soil security. Nat Plants. 2018;4:138–147. doi:10.1038/s41477-018-0108-y.

26. Xiao X, Zhang J, Wang H, Han X, Ma J, Ma Y, et al. Distribution and health risk assessment of potentially toxic elements in soils around coal industrial areas: A global meta-analysis. Sci Total Environ. 2020;713:135292. doi:10.1016/j.scitotenv.2019.135292.

27. Edwards CA, Lofty JR. The Effect of Direct Drilling and Minimal Cultivation on Earthworm Populations. J Appl Ecol. 1982;19:723. doi:10.2307/2403277.

28. Gerard BM, Hay RKM. The effect on earthworms of ploughing, tined cultivation, direct drilling and nitrogen in a barley monoculture system. J Agric Sci. 1979;93:147–155. doi:10.1017/S0021859600086238.

29. Chaney K, Hodgson DR, Braim MA. The effects of direct drilling, shallow cultivation and ploughing on some soil physical properties in a long-term experiment on spring barley. J Agric Sci. 1985;104:125–133. doi:10.1017/S0021859600043069.

30. Schjønning P, Rasmussen KJ. Soil strength and soil pore characteristics for direct drilled and ploughed soils. Soil and Tillage Res. 2000;57:69–82. doi:10.1016/S0167-1987(00)00149-5.

31. Koch HJ, Dieckmann J, Büchse A, Märländer B. Yield decrease in sugar beet caused by reduced tillage and direct drilling. Eur J Agron. 2009;30:101–109. doi:10.1016/j.eja.2008.08.001.

32. Suchet PA, Probst JL, Ludwig W. Worldwide distribution of continental rock lithology: Implications for the atmospheric soil CO_2 uptake by continental weathering and alkalinity river transport to the oceans. Global Biogeochem Cycles. 2003;17. doi:10.1029/2002GB001891.

33. Renforth P, Washbourne CL, Taylder J, Manning DAC. Silicate Production and Availability for Mineral Carbonation. Environ Sci Technol. 2011;45:2035–2041. doi:10.1021/es103241w.

34. Zani CF, Manning DAC, Abbott GD, Taylor JA, Cooper J, Lopez-Capel E. Diversified crop rotations and organic amendments as strategies for increasing soil carbon storage and stabilisation in UK arable systems. Front Environ Sci. 2023;11. doi:10.3389/fenvs.2023.1113026.

35. Bilsborrow P, Cooper J, Tétard-Jones C, Średnicka Tober D, Barański M, Eyre M, et al. The effect of organic and conventional management on the yield and quality of wheat grown in a long-term field trial. Eur J Agron. 2013;51:71–80. doi:10.1016/j.eja.2013.06.003.

36. KWS UK. WPB Elyann - Barley & Oats - Cereals - Products- KWS UK Ltd; 2021. Available from: https://mediamaster.kws.com/01_Products/UK_UnitedKingdom/Cereals/Variety-sheets/Oats-Data-Sheets/2021/WPB-Elyann-2021.22-2.pdf?_gl=1*q015ci*_ga*MTU3ODUwNTcyMC4xNjc3MTQ0NTgx*_ga_VWHSZDPV2B*MTY3NzE0NDU4MS4xLjEuMTY3NzE0NDY1MC4wLjAuMA.

37. Cooper J, Sanderson R, Cakmak I, Ozturk L, Shotton P, Carmichael A, et al. Effect of Organic and Conventional Crop Rotation, Fertilization, and Crop Protection Practices on Metal Contents in Wheat (Triticum aestivum). J Agric Food Chem. 2011;59:4715–4724. doi:10.1021/jf104389m.

38. Randall BAO. Dolerite-pegmatites from the Whin Sill near Barrasford, Northumberland. Proc Yorkshire Geol Soc. 1989;47:249–265. doi:10.1144/pygs.47.3.249.

39. Palandri J, Kharaka Y. A Compilation of Rate Parameters of Water-Mineral Interaction Kinetics for Application to Geochemical Modeling; 2004.

40. Murphy MR. Analyzing and Presenting pH Data. J Dairy Sci. 1982;65:161–163. doi:10.3168/jds.S0022-0302(82)82165-6.

41. Kelland ME, Wade PW, Lewis AL, Taylor LL, Sarkar B, Andrews MG, et al. Increased yield and CO_2 sequestration potential with the C_4 cereal *Sorghum bicolor* cultivated in basaltic rock dust-amended agricultural soil. Glob Chang Biol. 2020;26:3658–3676. doi:10.1111/gcb.15089.

42. Haque F, Santos RM, Chiang YW. Optimizing Inorganic Carbon Sequestration and Crop Yield With Wollastonite Soil Amendment in a Microplot Study. Front Plant Sci. 2020;11. doi:10.3389/fpls.2020.01012.

43. Manning DAC. Mineral stabilities in soils: how minerals can feed the world and mitigate climate change. Clay Miner. 2022;57:31–40. doi:10.1180/clm.2022.17.

44. Edwards DP, Lim F, James RH, Pearce CR, Scholes J, Freckleton RP, et al. Climate change mitigation: potential benefits and pitfalls of enhanced rock weathering in tropical agriculture. Bio Lett. 2017;13:20160715. doi:10.1098/rsbl.2016.0715.

45. Rea RS, Islam MR, Rahman MM, Nath B, Mix K. Growth, Nutrient Accumulation, and Drought Tolerance in Crop Plants with Silicon Application: A Review. Sustainability. 2022;14:4525. doi:10.3390/su14084525.

46. Agriculture and Horticulture Development Board. No-till: opportunities and challenges for cereal and oilseed growers; 2018. Available from: https://projectblue.blob.core.windows.net/media/Default/Imported%20Publication%20Docs/AHDB%20Cereals%20&%20Oilseeds/Soil/No-till%20opportunities%20and%20challenges%20for%20cereal%20and%20oilseed%20growers.pdf.

47. van Groenigen JW, Lubbers IM, Vos HMJ, Brown GG, Deyn GBD, van Groenigen KJ. Earthworms increase plant production: a meta-analysis. Sci Rep. 2014;4:6365. doi:10.1038/srep06365.

48. Bromfield SM. The effect of manganese-oxidizing bacteria and pH on the availability of manganous ions and manganese oxides to oats in nutrient solutions. Plant Soil. 1978;49:23–39. doi:10.1007/BF02149905.

49. Truog E. Soil Reaction Influence on Availability of Plant Nutrients. Soil Sci Soc Am J. 1947;11:305–308. doi:10.2136/sssaj1947.036159950011000C0057x.

50. Anda M, Shamshuddin J, Fauziah CI. Increasing negative charge and nutrient contents of a highly weathered soil using basalt and rice husk to promote cocoa growth under field conditions. Soil and Tillage Res. 2013;132:1–11. doi:10.1016/j.still.2013.04.005.

51. Luchese AV, Pivetta LA, Batista MA, Steiner F, da Silva Giaretta AP, Curtis JCD. Agronomic feasibility of using basalt powder as soil nutrient remineralizer. Afr J Agric Res. 2021;17:487–497. doi:10.5897/AJAR2020.15234.

52. Kemp SJ, Lewis AL, Rushton JC. Detection and quantification of low levels of carbonate mineral species using thermogravimetric-mass spectrometry to validate CO2 drawdown via enhanced rock weathering. Appl Geochem. 2022;146:105465. doi:10.1016/j.apgeochem.2022.105465.

53. Heenan DP, Chan KY, Knight PG. Long-term impact of rotation, tillage and stubble management on the loss of soil organic carbon and nitrogen from a Chromic Luvisol. Soil and Tillage Res. 2004;76:59–68. doi:10.1016/j.still.2003.08.005.

54. Hartemink AE, Barrow NJ. Soil pH - nutrient relationships: the diagram. Plant Soil. 2023;doi:10.1007/s11104-022-05861-z.

55. Oelkers EH. General kinetic description of multioxide silicate mineral and glass dissolution. Geochim Cosmochim Acta. 2001;65:3703–3719. doi:10.1016/S0016-7037(01)00710-4.

56. Kalinowski BE, Schweda P. Kinetics of muscovite, phlogopite, and biotite dissolution and alteration at pH 1?4, room temperature. Geochim Cosmochim Acta. 1996;60:367–385. doi:10.1016/0016-7037(95)00411-4.

57. Bonneville S, Smits MM, Brown A, Harrington J, Leake JR, Brydson R, et al. Plant-driven fungal weathering: Early stages of mineral alteration at the nanometer scale. Geology. 2009;37:615–618. doi:10.1130/G25699A.1.

58. EC. Commission Regulation (EC) No 1881/2006 of 19 December 2006 setting maximum levels for certain contaminants in foodstuffs (Text with EEA relevance); 2006. Available from: http://data.europa.eu/eli/reg/2006/1881/oj/eng.

59. Shay J. Israel- Heavy Metals limits in Foodstuffs. Tel Aviv: United States Department of Agriculture, Foreign Agricultural Service; 2022. IS2022-0008.

Figure 1

Click here to access/download;Figure;Fig1.tiff

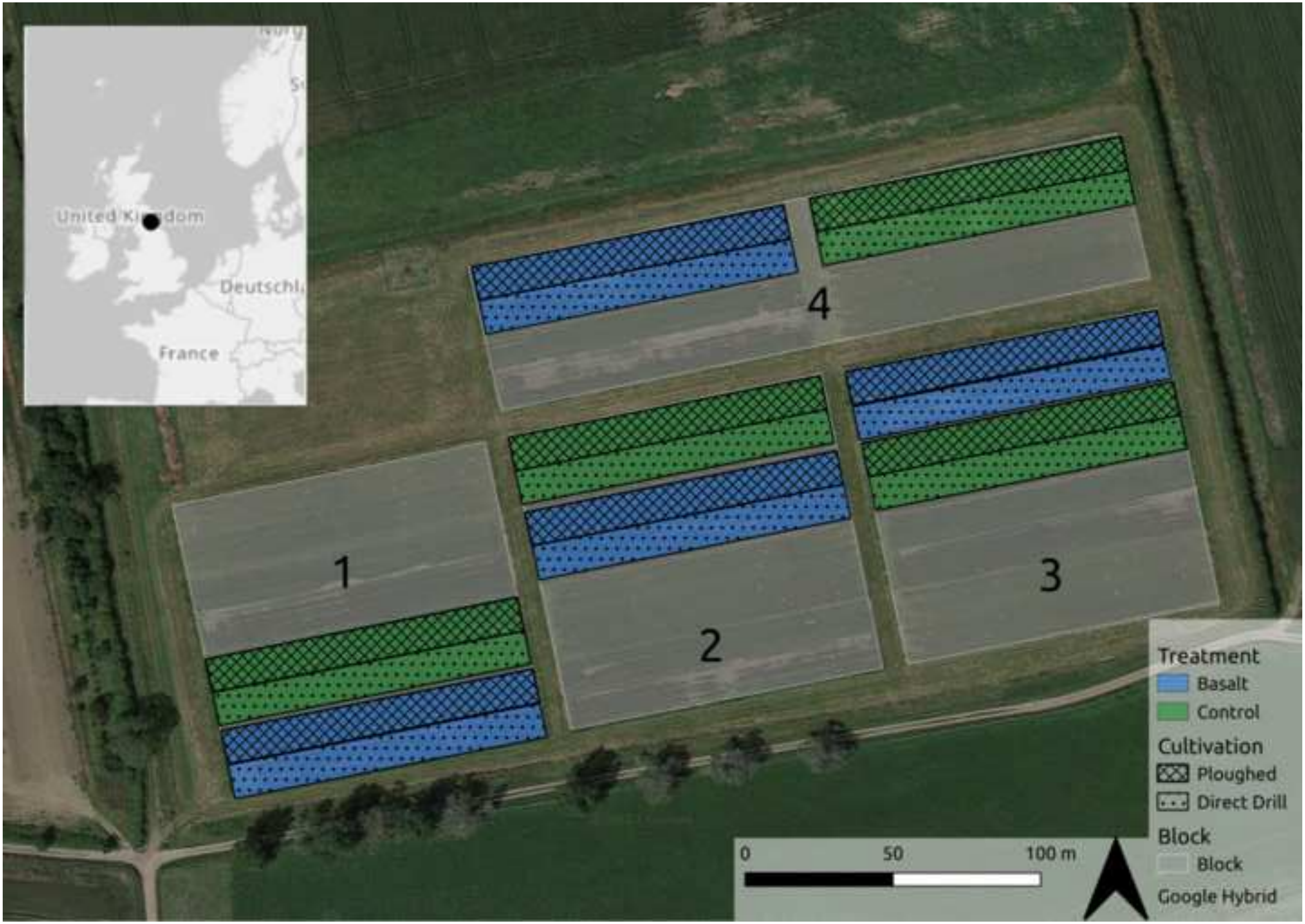

Figure 2

Click here to access/download;Figure;Fig2.tif

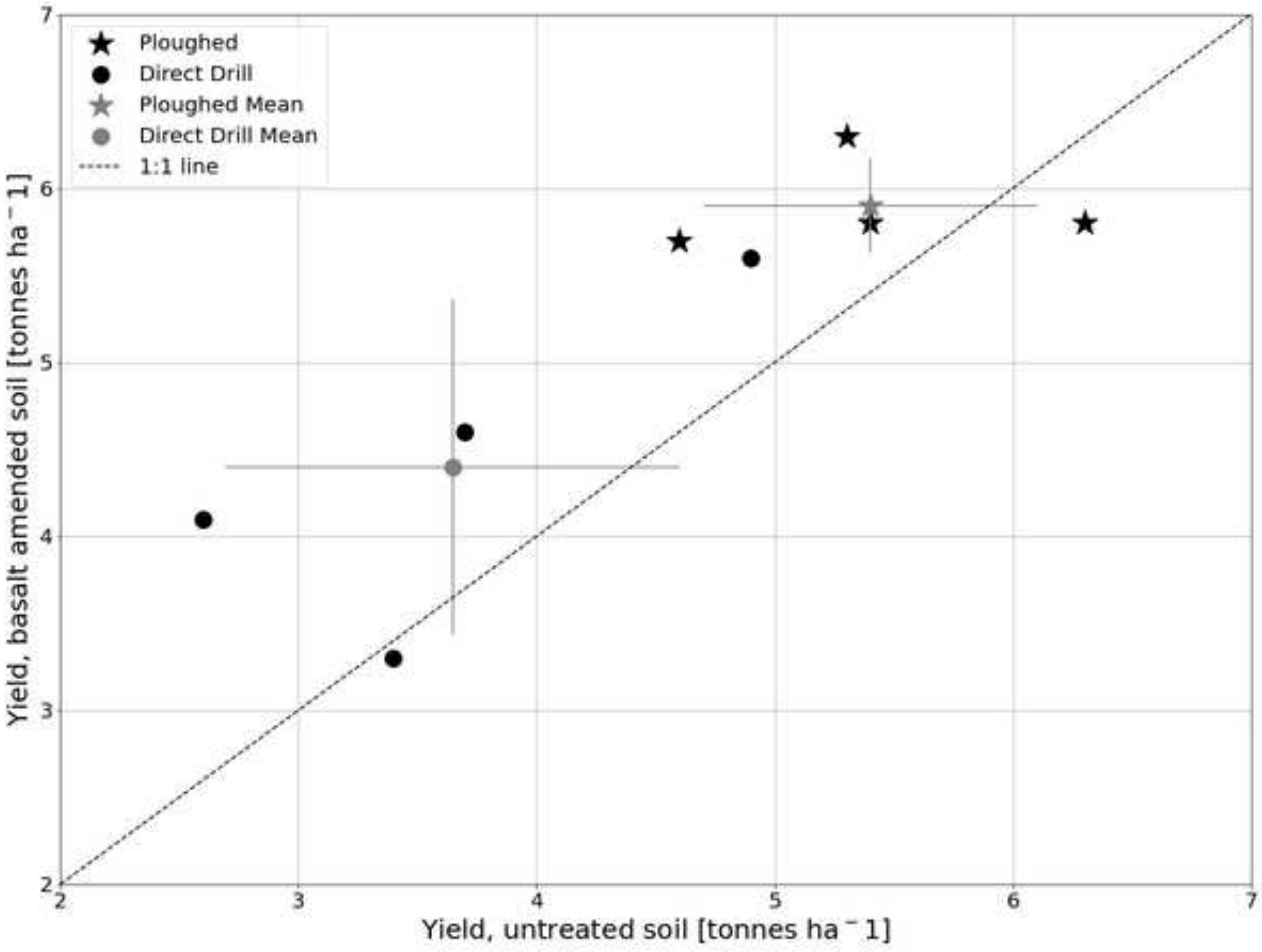

Figure 3

Click here to access/download;Figure;Fig3.tif

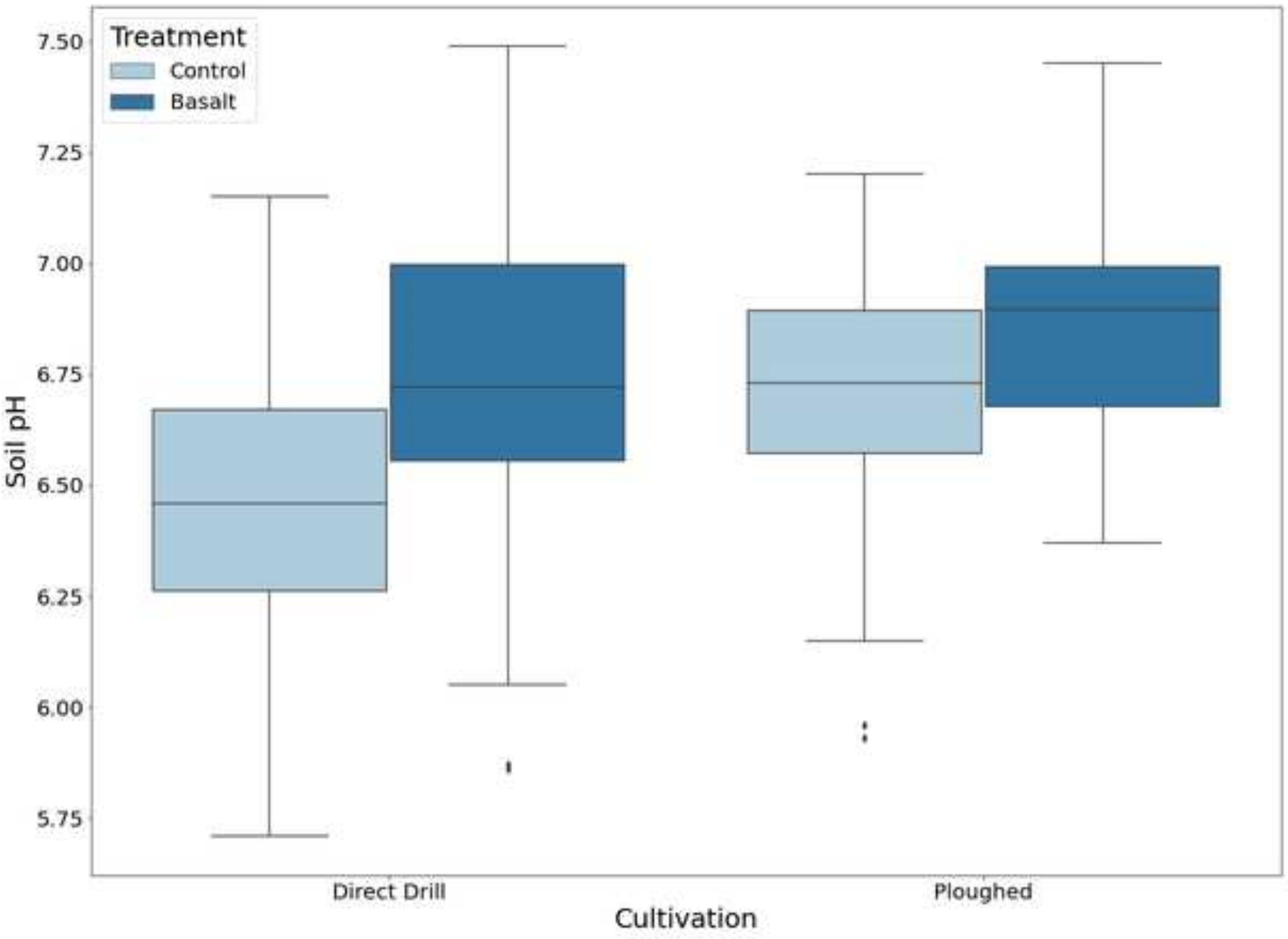

Click here to access/download
Supporting Information - Compressed/ZIP File Archive
SupportingInformation.zip
