# Supplementary material for: Initial agronomic benefits of enhanced weathering using basalt: A study of spring oat in a temperate climate
Source: PLoS One. 2024 Mar 27;19(3):e0295031. doi: 10.1371/journal.pone.0295031 (PMC10971544; doi:10.1371/journal.pone.0295031)
Supplement: Attachment — Submitted filename: ResponseToReviewers.pdf [file pone.0295031.s005.pdf]

Thank you very much for the comments on our manuscript. We've amended the manuscript to include points raised in the comments where possible and given our reasoning as to why we haven't implemented comments where these amendments were not possible. Please see responses to comments in italic.

Journal Requirements:

When submitting your revision, we need you to address these additional requirements.

1. Please ensure that your manuscript meets PLOS ONE's style requirements, including those for file naming. The PLOS ONE style templates can be found at https://journals.plos.org/plosone/s/file?id=wjVg/PLOSOne_formatting_sample_main_body.pdf and https://journals.plos.org/plosone/s/file?id=ba62/PLOSOne_formatting_sample_title_authors_affiliations.pdf

Several changes were made throughout the manuscript to comply with the above requirements.

2. In your Methods section, please provide additional information regarding the permits you obtained for the work. Please ensure you have included the full name of the authority that approved the field site access and, if no permits were required, a brief statement explaining why.

The Nafferton Research farm at which this study was conducted is owned by the University of Newcastle and as such no permissions were required.

A sentence has been added in the site description.

3. Thank you for stating the following financial disclosure:
"The study design and yield data collection was conducted by Newcastle University, independent of funders of the project.
All other data collection and analysis was funded by UNDO Carbon ltd.
Decision to publish and preparation of the manuscript was done in collaboration between Newcastle University and UNDO Carbon ltd."

Please state what role the funders took in the study. If the funders had no role, please state: "The funders had no role in study design, data collection and analysis, decision to publish, or preparation of the manuscript."
If this statement is not correct you must amend it as needed.
Please include this amended Role of Funder statement in your cover letter; we will change the online submission form on your behalf.

The funder, UNDO Carbon Ltd., was involved in the data collection, analysis, decision to publish and preparation of the manuscript. Hence above statement has been modified to make this clearer:

"The study design and yield data collection was conducted by Newcastle University, independent of funders of the project.
The funders (UNDO Carbon ltd) were involved in all other data collection (including soil

sampling and plant tissue sampling) and analysis, conducted in collaboration with Newcastle University.
Decision to publish and preparation of the manuscript was done in collaboration between Newcastle University and UNDO Carbon ltd."

4. Thank you for stating the following in the Competing Interests section:
"Kirstine Skov, Jez Wardman, Matthew Healey, Amy McBride, Tzara Bierowiec, Ifeoma Edeh, Melissa J. Murphy, Ryan Pape, Will Turner, Peter Wade and Xinran Liu are presently or were recently employed in the Science and Research department and may hold options in UNDO Carbon ltd.
Mike Kelland is an independent consultant for UNDO Carbon ltd.
Jim Mann is the founder and CEO of UNDO Carbon ltd.
David Manning is part of UNDO carbon ltd.s scientific advisory board.
Julia Cooper, Dave George and Yit A. Teh declare no conflicts of interest."

"This does not alter our adherence to PLOS ONE policies on sharing data and materials."

Please confirm that this does not alter your adherence to all PLOS ONE policies on sharing data and materials, by including the following statement: "This does not alter our adherence to PLOS ONE policies on sharing data and materials." (as detailed online in our guide for authors http://journals.plos.org/plosone/s/competing-interests). If there are restrictions on sharing of data and/or materials, please state these. Please note that we cannot proceed with consideration of your article until this information has been declared.

Please include your updated Competing Interests statement in your cover letter; we will change the online submission form on your behalf.

5. We note that you have indicated that data from this study are available upon request. PLOS only allows data to be available upon request if there are legal or ethical restrictions on sharing data publicly. For more information on unacceptable data access restrictions, please see
http://journals.plos.org/plosone/s/data-availability#loc-unacceptable-data-access-restrictions.

In your revised cover letter, please address the following prompts:

a) If there are ethical or legal restrictions on sharing a de-identified data set, please explain them in detail (e.g., data contain potentially sensitive information, data are owned by a third-party organization, etc.) and who has imposed them (e.g., an ethics committee). Please also provide contact information for a data access committee, ethics committee, or other institutional body to which data requests may be sent.

b) If there are no restrictions, please upload the minimal anonymized data set necessary to replicate your study findings as either Supporting Information files or to a stable, public repository and provide us with the relevant URLs, DOIs, or accession numbers. For a list of acceptable repositories, please see
http://journals.plos.org/plosone/s/data-availability#loc-recommended-repositories.

We will update your Data Availability statement on your behalf to reflect the information you provide.

See next point

6. We note that you have stated that you will provide repository information for your data at acceptance. Should your manuscript be accepted for publication, we will hold it until you provide the relevant accession numbers or DOIs necessary to access your data. If you wish to make changes to your Data Availability statement, please describe these changes in your cover letter and we will update your Data Availability statement to reflect the information you provide."

The data is shared in the public repository Zenodo: 10.5281/zenodo.8158681

7. We note that you have included the phrase "data not shown" in your manuscript. Unfortunately, this does not meet our data sharing requirements. PLOS does not permit references to inaccessible data. We require that authors provide all relevant data within the paper, Supporting Information files, or in an acceptable, public repository. Please add a citation to support this phrase or upload the data that corresponds with these findings to a stable repository (such as Figshare or Dryad) and provide and URLs, DOIs, or accession numbers that may be used to access these data. Or, if the data are not a core part of the research being presented in your study, we ask that you remove the phrase that refers to these data.

Sections where 'data not shown' were written have been modified.

8. We note that Figure 1 in your submission contain map/satellite images which may be copyrighted. All PLOS content is published under the Creative Commons Attribution License (CC BY 4.0), which means that the manuscript, images, and Supporting Information files will be freely available online, and any third party is permitted to access, download, copy, distribute, and use these materials in any way, even commercially, with proper attribution. For these reasons, we cannot publish previously copyrighted maps or satellite images created using proprietary data, such as Google software (Google Maps, Street View, and Earth). For more information, see our copyright guidelines: http://journals.plos.org/plosone/s/licenses-and-copyright.

We require you to either (a) present written permission from the copyright holder to publish these figures specifically under the CC BY 4.0 license, or (b) remove the figures from your submission:

a. You may seek permission from the original copyright holder of Figure 1 to publish the content specifically under the CC BY 4.0 license.

We recommend that you contact the original copyright holder with the Content Permission Form (http://journals.plos.org/plosone/s/file?id=7c09/content-permission-form.pdf) and the following text:
"I request permission for the open-access journal PLOS ONE to publish XXX under the Creative Commons Attribution License (CCAL) CC BY 4.0 (http://creativecommons.org/licenses/by/4.0/). Please be aware that this license allows unrestricted use and distribution, even commercially, by third parties. Please reply and provide explicit written permission to publish XXX under a CC BY license and complete the

attached form.”

Please upload the completed Content Permission Form or other proof of granted permissions as an "Other" file with your submission.

In the figure caption of the copyrighted figure, please include the following text: “Reprinted from [ref] under a CC BY license, with permission from [name of publisher], original copyright [original copyright year].”

b. If you are unable to obtain permission from the original copyright holder to publish these figures under the CC BY 4.0 license or if the copyright holder’s requirements are incompatible with the CC BY 4.0 license, please either i) remove the figure or ii) supply a replacement figure that complies with the CC BY 4.0 license. Please check copyright information on all replacement figures and update the figure caption with source information. If applicable, please specify in the figure caption text when a figure is similar but not identical to the original image and is therefore for illustrative purposes only.
The following resources for replacing copyrighted map figures may be helpful:

USGS National Map Viewer (public domain): http://viewer.nationalmap.gov/viewer/
The Gateway to Astronaut Photography of Earth (public domain): http://eol.jsc.nasa.gov/sseop/clickmap/
Maps at the CIA (public domain): https://www.cia.gov/library/publications/the-world-factbook/index.html and https://www.cia.gov/library/publications/cia-maps-publications/index.html
NASA Earth Observatory (public domain): http://earthobservatory.nasa.gov/
Landsat: http://landsat.visibleearth.nasa.gov/
USGS EROS (Earth Resources Observatory and Science (EROS) Center) (public domain): http://eros.usgs.gov/#
Natural Earth (public domain): http://www.naturalearthdata.com/

It’s unfortunate that PLOS ONE doesn’t allow open source materials from Google images, as we were not able to find any other source with a resolution that allowed for a sensible use as background image. We’ve kept the openstreetview background in the figure, as it’s our understanding that this complies with the CC BY 4.0 license.

Academic Editor's Comments:

- Please avail the details requested by reviewers (as much as possible).
- A closer look at the manuscript suggests that most of the items provided as supplementary files should have been supplied in the main manuscript file to provide a better understanding of the study. Please consider this while performing your revision.

Given Reviewer #1 request to have more info on how the different analyses of the basalt relate to each other, we’ve included the basalt oxide and elemental composition in the main manuscript. For the other documents in the supplementary material it is our opinion that the structure of the paper and the storyline is kept more intact with those staying in the supplementary material.

[Note: HTML markup is below. Please do not edit.]

Reviewers' comments:

Reviewer's Responses to Questions

Comments to the Author

1. Is the manuscript technically sound, and do the data support the conclusions?

The manuscript must describe a technically sound piece of scientific research with data that supports the conclusions. Experiments must have been conducted rigorously, with appropriate controls, replication, and sample sizes. The conclusions must be drawn appropriately based on the data presented.

Reviewer #1: Partly

Reviewer #2: Yes

---

2. Has the statistical analysis been performed appropriately and rigorously?

Reviewer #1: I Don't Know

Reviewer #2: Yes

---

3. Have the authors made all data underlying the findings in their manuscript fully available?

The PLOS Data policy requires authors to make all data underlying the findings described in their manuscript fully available without restriction, with rare exception (please refer to the Data Availability Statement in the manuscript PDF file). The data should be provided as part of the manuscript or its supporting information, or deposited to a public repository. For example, in addition to summary statistics, the data points behind means, medians and variance measures should be available. If there are restrictions on publicly sharing data—e.g. participant privacy or use of data from a third party—those must be specified.

Reviewer #1: Yes

Reviewer #2: Yes

---

4. Is the manuscript presented in an intelligible fashion and written in standard English?

PLOS ONE does not copyedit accepted manuscripts, so the language in submitted articles must be clear, correct, and unambiguous. Any typographical or grammatical errors should be corrected at revision, so please note any specific errors here.

Reviewer #1: Yes

Reviewer #2: Yes

---

5. Review Comments to the Author

Please use the space provided to explain your answers to the questions above. You may also include additional comments for the author, including concerns about dual publication, research ethics, or publication ethics. (Please upload your review as an attachment if it exceeds 20,000 characters)

Reviewer #1: This study provides one of the first field trial results of basalt rock use for enhanced weathering (EW) in the agricultural context. It is a short-term small-plot studies, so not quite a true "field study", but still has some value in taking the research forward from conceptual to deployment. The study does have some shortcomings in terms of data collected and results interpretation, and below these are discussed with suggestions for improvement and re-consideration.

Lines 11-12: interesting to cite such old studies on the suggestion of basalt as a soil mineral. What happened that this pratice has not become widespread? Can a bit more about the history of basalt as a soil amendment be said to explain the current state and why CDR is needed as an added incentive?

The application of silicate rocks such as basalt as EW amendments to cropland offer a long list of potential agronomic benefits [7–9]. Studies dating back to the 1960's [10,11] and even 1930's [12,13] have researched the benefits of applying crushed silicate rock, specifically basalt, to agricultural and forest soils. However, some studies of volcanic rock dust have shown little or no agronomic benefit (e.g. Ramezanian, A., Dahlin, A.S., Campbell, C.D., Hillier, S., Mannerstedt-Fogelfors, B. & Öborn, I. 2013. Addition of a volcanic rockdust to soils has no observable effects on plant yield and nutrient status or on soil microbial activity. Plant & Soil, 367, 419–436). In this particular case, the rock used was not described, and, although it appears not to be basaltic on the basis of its high iron content, the study may have been influential in deterring interest in the use of crushed basalt.

However, clear benefits for plant growth have been shown in more rigorous experiments [41], in which the crushed rock was well defined as basaltic on the basis of its chemical and mineralogical composition. Mafic rocks, such as basalt, are defined mineralogically as containing pyroxene and plagioclase-group minerals (Wilson, 1989) and are defined chemically as containing <52 wt.% SiO_2 and < 5 wt.% alkali metal oxides (Na_2O and K_2O) (Tas et al., 1986). As a result of these definitions, the composition of mafic rocks can vary considerably between and with location and geological history (Wilson, 1989; Lewis et al., 2021). This variability makes it extremely important to characterise the mineralogy and chemistry of each rock used within experiments.

Until very recently, interest in the agronomic benefits of basalt has been limited; the use of this rock in enhanced rock weathering studies has provided a new and urgent incentive for rigorous trials that investigate the effects on crop growth. Basalt is rich in minerals that

release macro- (e.g., calcium, magnesium, silicon, potassium, phosphorus, sulfur, but excluding nitrogen) and micro-nutrients (e.g., copper, iron, manganese, molybdenum and zinc) during dissolution that are essential for plant growth [4–6]. Crushed silicate rocks have been studied with respect to underestimated potassium and micronutrient deficiencies, following agricultural soil nutrient stripping [9]. Some of these micronutrients are redox-active which makes them important cofactors in enzymatic, metabolic and cellular processes [14]. Additionally, these micronutrients are actively involved in nitrogen fixation and photosynthesis [15,16].

Line 56: it says "basalt does not necessarily contain the mineral olivine". Coincidently, I just spoke to a geologist yesterday who said the opposite, that some basalts have olivine. The compositional variation of basalt is something concerning about several studies referring to basalt as if being a well defined material. Can a bit more discussion be added to the introduction to contrast how variable basalt can be, and what makes a basalt suitable for the intended application?

We have removed the secondary sentence about olivine, as the main point is that basalt may have nickel levels exceeding EU inorganic soil improver limits, although this is not the case for the basalt studied here.

Regarding the variability of basalt, a section on this has been added further up in the introduction, where benefits of basalt application is discussed. (Lines 19-25)

Lines 60-72: this paragraph seems a bit disconnected to the rest of the introduction. It can be put into better context with connecting start and end sentences.

Start and end sentences have been added.

Line 76: the term "grant access to their land to apply it" suggests that EW is a technology in the hand of third parties who seek permission of landowners to apply, which is kind of how the MRV market has been shaping up... Wouldn't it be better for EW to be a technology that farmers want to implement in their fields out of their own interest? If there agronomic benefits, and agronomic role, then agronomic practices such as amendment rates based on standardized recommendations and amendment methods that are optimized for the crop should be used, rather than for maximizing carbon removal certificates. Is this the direction that the authors are looking into in terms of justifying basalt use based on agronomic benefits?

The sentence has been modified to: 'However, the availability of crushed rock alone is insufficient if landowners are not inclined to grant access to their land for third parties to apply it or actively apply basalt to their land themselves.

Line 105: how was the amendment rate of 18.86 tonnes per hectare selected? It is a reasonable rate (i.e., not as high as some studies suggest), so it would be especially useful to know what the rationalle was to better guide future studies.

The sentence has been modified, see response to next comment

Lines 105 to 108: it is confusing. If the plots were ploughed at the end of March, how was the basalt incorporated into the soil upon broadcast spreading? Not clear how the mineral was mixed into the soil, was it lightly tilled after spreading?

It was described in line 110-111 that incorporation of basalt both for the ploughed and direct drill plots was only done during seeding. The lines 105-112 have been rearranged, so field management practices are reported chronologically. It's also been specified that no other incorporation strategies were used:

The ploughed fields were tilled to a depth of 15-30 cm at the end of March, followed by a press within three days (using Simba Unipress). Basalt was applied on Apr 12th 2022 using a conventional agricultural lime spreader. The target application rate was set to 20 tonnes ha^{-1}, as this is the maximum the commercial spreader can do in a single pass, the actual mean spreading rate was determined to be 18.86 tonnes ha^{-1}. The ploughing was done prior to basalt application, hence the basalt was exclusively incorporated to the seeding depth across both cultivation types. No further efforts were made to incorporate the basalt into the soil, as commercial methods were employed to ensure the process accurately represented farm scale operations.

Line 126: <2 mm?

Thank you for making us aware of the error, it has been amended accordingly.

Line 128: what were the SOC and SIC contents of the soil?

SOC and SIC were not determined on the soil samples taken as part of this study.

Information on SOC from Zani et al., 2023 has been added to the site description:
The soil is slightly acidic (average pH in 2011 of 6.3) [35], with an average soil organic carbon stock of 60.64 ±0.9 MgC ha^{-1}.

Table 1 and Table S2: in how much agreement are the mineral composition and the chemical composition, stoichiometry-wise?

Thanks for the suggestion, this is great and as a community interested in rock chemistry and mineralogy, it sounds sensible that all types of rock amendments used in similar studies should be evaluated in this way.

We have taken your suggestion, and we have compared the agreement between the observed (XRF and ICP-OES) and calculated (from XRD data) wt.% for major elements within the rock (Na, Ca, K, Al, Mg, Fe, Ti, Si - excluding O as this was not independently measured with these methods). We found, first of all, when comparing our XRF and ICP-OES, that these results agree well - with up to a 0.42 wt.% difference between the two measurement techniques for a given element (equivalent to a 7.1% percentage difference).

Most of the minerals we have identified (all except calcite and quartz - though there may be minor mineral substitutions in these minerals also) form a solid solution between Ca and Na

(andesine and augite), as well as the Al and Si (e.g. notably illite, chlorite and smectite - and to a lesser extent andesine and augite) end members.

As a result, it is not simple to compare the mineralogy to the chemical data.

We found that the datasets best matched each other when Na and Al were limited in the solid solutions (e.g. when the mineral formula contains (Ca, Na) - Ca = 1 and Na = 0; when the mineral formula contains (Al, Si) - Al = 0, Si = 1). This is when the calculated (mineralogical) Na and Al data best fit the measured chemical data. This result agrees with detailed mineralogical analysis that was conducted in Randall et al. (1989) - who have identified high-Ca augite (with mineral analyses showing very little Na) and high-Si secondary phases (e.g. chlorite) relative to Al.

When assuming that no Al was present in Al and Si solid solutions (with the exception of andesine, where Ca-Na-Al-Si concentrations are all interdependent) we found that XRF/ICP-OES concentrations were less than <0.74 wt.% in measured difference (e.g. ICP-OES for Mg (converted from MgO) = 2.99 wt.%, results = 3.73 wt.%) and up to a 22.1% percentage difference (between all elements).

If we consider an 'equal distribution' between solid solutions (e.g. (Si,Al) - Si = 0.5 and Al = 0.5; (Ca, Na) - Ca = 0.5 and Na = 0.5), we obtain measured differences of up to 6.35 wt.% and with percentage differences between the two documents of up to 63.93%.

We also considered using a CIPW-norm calculation, but this can be less reliable due to the presence of clay minerals within this rock - which may overestimate the amount of quartz present. We chose to omit any numerical differences in results in the manuscript text because the data was based simply on modelled assumptions.

As a result, we have added the following text to the 'Basalt' subsection (Lines 194-196):

"Chemical analyses (ICP-OES and XRF) data are consistent with each other (< 0.42 wt.% difference). The chemical analyses are also comparable to the XRD data, when comparing mineral stoichiometry to both methods of chemical analysis."

Table 1: "fast" is relative, is a term like "faster" more appropriate here?

'Fast' and 'slow' have been amended to 'faster' and 'slower' in Table 1 and throughout the text when 'fast' and 'slow' weathering minerals are referred to.

Table 1: as this article is about the agronomic benefits of basalt, and part of those agronomic benefits would come from the release of P and K plant nutrients (from Table S2), the question is where are those elements present in the mineralogy? In addition, what is the mineral assemblage of these phases in the particles? SEM-EDX or EPMA study of basalt is something largely lacking from basalt studies. It is not clear which of the mineral phases would react first, under what extent of weathering it could be expected that the plant benefiting elements would be released, or even what secondary minerals may form during the weathering sequence that could passivate the remaining minerals from further weathering. Considering that this is a single-growth season study, it is even more important to understand what part of basalt can react within a few months.

We have broken this question down into several components:

Where is the P and K?

We understand the reviewers' concern about 'where the P and K' are constrained. We have made reasonable scientific assumptions about where K has come from.

We provided generalised stoichiometric formulas for the minerals in Table 1. Here, it is possible to identify K in the illite-mica phase and we make reference to this in the discussion section of the paper.

With regards to P, it may be that the P is identified as trace apatite, in concentrations too small to be identified by XRD (using this XRD analysis system, at < 1wt.%) - based on the amount of P present in the rock, 0.1280 wt.%, and the scaling factor of 5.38 to convert between P to apatite, the concentrations of apatite would be 0.7 wt.% apatite - which is too small for us to identify with XRD. We saw no significant difference in P as a result of basalt amendment (p < 0.320, Table 5) - so we did not discuss the presence of P as, while it is an essential nutrient, there were no observations of significant differences in this study.

We could add the following sentence to the Nutrient uptake section (Lines 427-431):

The essential plant nutrient, phosphorus, is present in the Divet Hill basalt at concentrations of 0.1280 wt.% (Table 3) although we did not detect any calcium phosphate apatite in the rock. At this concentration of phosphorus, apatite would be present at a concentration of 0.7 wt.%, which is not likely to be resolved using XRD. Furthermore, we did not identify a significant difference in the phosphorus concentration in the plant grain or tissue as a result of basalt amendment (Table 5).

Concern with SEM-EDX and EMPA

We understand the reviewers' concern regarding the lack of analytical techniques analysing the mineral chemistry of the mineral phases within the basalt.

While this certainly is a worthwhile task to improve our (as an enhanced weathering community) understanding of weathering in soils, it is considerable work to image and determine the chemistry of weathered basalt grains (due to the complexity of minerals) - particularly in a non-biased and statistically significant way. Furthermore, although basalt weathers reasonably fast compared to other common rock types, it is still difficult to observe definitive signs of weathering in basalt grains.

Which minerals will react first?

It is not possible to determine 'which mineral phases would react first' as they may well be weathering all together, as predicted from modelling presented in Kelland et al 2020 (Figure 5, where all minerals start dissolving, but some (olivine and diopside) are dissolving rapidly and other minerals are dissolving slower) and Lewis et al 2022 (Figure 7, where a variety of minerals are all dissolving at once for any given sample).

We have no evidence to determine which minerals were weathering first specifically from our study, and it would not be scientifically sensible to discuss this. There are plenty of published experiments which have used basalt as a rock amendment and have not provided direct evidence for which mineral components were expected to weather. However, in Table 1 we have provided the quantitative XRD analysis as well as the dissolution rate constants for both acidic and neutral reaction mechanisms, and make reference to this table throughout our discussion section.

In the absence of being able to directly measure which minerals are weathering, we have tried to predict which mineral phases may be weathering faster, the dissolution rates are discussed in the sections relating to plant nutrient uptake and soil pH. To make these links clearer, we have edited the manuscript to provide a better explanation for the possible minerals which are contributing to the different mineral rates.

We have already made reference to the location of K in the discussion section (see lines 403-408). For Ca and Mg, we have amended the following sentence:

'The Divet Hill basalt contains calcium (CaO: 8.97 wt.%) and magnesium (MgO: 5.32 wt.%) (Table 2), and both cations are present in the faster dissolving silicate phases (e.g. augite and amphibole - Table 1). However, calcium is also present in the faster dissolving calcite (Table 1 - at least 30,000-fold faster dissolution than augite and amphibole), which is thought to be the main source of calcium during the first growing season.

It is beyond the scope of this study to determine to what extent the basalt has weathered, and this is clear from our objectives which we have specified to be focused on first year agronomy benefits.

Secondary phase precipitation

Based on the datasets we have provided in our paper, it is not possible to determine the precipitation of secondary phases. This is purely an agronomic paper, focusing on the first growing season following basalt application, with a particular focus on the crop and not the soil (with the exception of soil pH, which is a pivotal agronomic parameter). Any mention of secondary phase precipitation would be purely speculative - since we did not collect pore water from the plot, it was not possible to determine saturation indices to predict the precipitation of secondary phases nor did we conduct any sequential extractions to identify the precipitation of e.g. amorphous Si, or Al/Fe-hydroxides. Even if we did conduct this analysis, we do not know where these secondary phases would precipitate - in the soils or on basalt grains. If precipitating in the soil, they will not passivate basalt particle surfaces.

Figure 2: interesting that one plot each of direct drill and ploughed (i.e., one circle and one star) are below the 1:1 line. Is there any spatial relationship (looking at Figure 1) in the plots the performed better and performed worse? For instance, the blue plots of block 1 are closer to trees, and trees tend to absorb nutrients from neighboring crops (in croplands, plants at

the edge near tree lines always grow less). Also, is there any sense about the direction of run-off across the field? Also, was water infiltration rates equal in each block?

The two points below the 1:1 line in Figure 2 are from block 3 and 2, for ploughed and direct drill plots respectively. Hence from the two blocks where the edge effect must be assumed to be the lowest, but not replicated across both cultivation types in both blocks. The two points falling below the 1:1 line could be natural variability or due to some parameter that we didn't look into. The spatial variability between the four blocks is accounted for in the linear mixed effect model by including the block term as a random effect term.

Unfortunately, there is no information about the infiltration rates between the blocks. There is a slight slope on the west to east axes of the field, ranging from 110-113 meter above sea level on the western side to 100-103 meter above sea level on the eastern side. The distance from the western to the eastern edge of the field is 350 meter, resulting in a 2.8% slope parallel to the plots.
This sentence is added to the 'Site description' in the methods section:
The field is slightly sloping in the west to east direction of the field, with a slope corresponding to 2.8% parallel to the plots.

Figure 3: considering that the box plots have rather large whiskers, three boxes that are very much overlapping, and even some outlier points, it is surprising that Table 3 reports p values <0.001 for both cultivation and basalt effects. Likewise so many p values in Table 3 are <0.001 yet the values in Table 2 are not very different between the plots. Here also the question about the effect of spatial variability versus treatment effect comes in.

Yes, the standard deviations in the control and basalt amended groups are rather large in comparison to the difference in the mean between the same (Figure 3 and Table 4). It is not uncommon for soil parameters to exhibit very large heterogeneity (which was the main reason for the large number of soil sub samples within each plot, n = 16 for the pH determination). Likewise, for the rest of the parameters listed in Table 4, the relatively low (but in an agricultural context common) number of plot replicates (n = 4), combined with the natural variability in crop nutrients, results in rather 'noisy' data, with a high variability to mean ratio. Nevertheless, we believe that we've carried out rigorous statistical testing of the presented dataset, with the statistically significant differences reproduced using a two-way ANOVA including the block term (see for example Schielzeth et al., 2020). Of the two methods (linear mixed effect model (LMEM) and two-way ANOVA including the block term), the LMEM was preferred for this manuscript, as previous studies have identified this method as particularly robust in relation to the above mentioned common variability in 'real datasets' (Schielzeth et al., 2020). There is an effect of the spatial variability between the blocks, which has been accounted for by including the block term as a random effect term in the LMEM. It's clear that there is spatial variability between the different blocks that can't be explained through the parameters investigated in this study. We believe that excluding the block term from any statistical test of this dataset would not do the experimental design justice. However, the main factors of interest are the basalt treatment and cultivation type, hence the reason these have been included as fixed effect terms.

The method sub section 'Statistical analysis' has been modified to include:
"A linear mixed effect model (LMEM) was used to evaluate whether the effect of basalt

treatment and cultivation type was statistically significant. The treatment and cultivation factors were included as fixed effect terms and the block factor as the random effect term, ***the latter to account for the spatial heterogeneity between the blocks.****"*

General comment: the only sign of weathering presented is the pH effect. This being an EW study, why were no measurements that are under consideration for MRV reported, such as: calcimetry, soil TGA, soil XRD, soil water alkalinity, soil water electrical conductivity, soil CO2 gas, soil isotope ratios, etc.

Indeed, a complete study involving all the above mentioned parameters would be extremely desirable for a MRV study. This is a study of EW, but with the focus of demonstrating agronomic benefits in a temperate climate, hence the focus on yield, tissue and grain nutrients, underlined by measurements of soil pH. Furthermore, the authors would like to argue that the pH effect is not the only sign of weathering reported in this study. In fact, given that the products from the mineral dissolution of basaltic rock will be in the aqueous soil phase and hence available for plant uptake, the difference in yield between the control and the basalt amended plots is very likely to be the first observable sign of weathering in any EW study conducted in an agricultural setting. This is further supported by the observed statistically significant effect of basalt amendment on tissue calcium and potassium, as well as grain potassium. In particular, the increase in potassium concentration in plant tissue is not likely to be due to an increase in pH.

Discussion section: how much of the effect of basalt amendment on yield is due to the pH effect versus other EW effects? Considering how slowly basalt has been reported to weather, it can be expected that the weathering extent in the study period was small. So other than buffering soil pH, how can it be deemed that basalt in this study performed better than conventional liming with limestone? Plants tend to have higher yield and better nutrient uptake when grown in soils under optimal pH, so could this largely explain the results reported? If not, then how to make a conclusive case that basalt's effects were more than just its liming effect? In hindsight, this study would have benefited from an extra set of plots with conventional liming used.

Yes, a comparison with limed plots would have been interesting. It's very likely that the agronomic first year effects of using crushed silicate rock is mostly connected to the stabilising effect on soil pH. We consider this a major asset of crushed silicate rock as a product, as the appeal of the product will be higher if farmers can use it instead of existing products, rather than as an additional supplement to the soil.

Lines 243 to 245: This statement highlights how the results are somewhat surprising. This calls for a more definitive understanding as to why the results were better than expected. The basalt used was certainly rather coarse, which would suggest even slower weathering than other studies have suggested. So why were the results so good? Perhaps part of the "amorphous" content of the mineral was made up of free alkaline oxides that reacted quickly? Perhaps this andesine-rich basalt (which from literature seems to not be so common) is more reactive than typical basalts? Is the rock used actually basaltic andesite rather than basalt? Some of this is commented in later lines, but without a definitive conclusion other than the suggestion that the basalt used may have been more reactive than other basalts.

The sentence directly following these lines was somewhat unnuanced, as it's not the case that we wouldn't expect any positive effects of basalt application in the first year, rather that we don't want to give the impression that we think you can always expect a yield result that is 10-20% higher compared to fields where no basalt has been applied. That sentence has been modified accordingly. That whole section was included to discuss the potential of environmental extremes (such as the very dry 2022 growing season in NE England) to amplify the positive effects of soil amendments.

With regard to the comment on the type of rock used, we have confirmed that, based on oxide chemistry, the Divet Hill material is classified as a basalt and not a basaltic andesite (e.g. using the TAS diagram (Le Bas et al, 1986)) and have added this to the basalt characterisation section.

We have added the following text to the document under the Methods - Basalt section (Lines 198 - 200):

"Using the TAS classification (Le Bas et al, 1986) and oxide data from XRF, the Divet Hill material is classified as having a chemical composition of a basalt."

Regarding the amorphous content of the basalt: we have added an additional line clarifying that the amorphous phase has not been identified as basaltic glass, and have amended our text in the Methods - Basalt section (Lines 190-195):

"In the absence of scanning electron microscopy-energy dispersive spectroscopy (SEM-EDS), it was not possible to determine the chemical composition of the amorphous phase(s). However, Randall (1989) states that talc occurs in association with pyroxene weathering, and this and other clay minerals, if poorly crystalline, may contribute to the amorphous component."

Line 274: here is more information about the basalt amendment that lacked earlier. Still not clear if the basalt was evenly tilled into the soil during this seeding process.

See amendments made to line 128-135 above.

Lines 293 to 294: this statement about the reference is not correct. The cited study states: "At the end of the growth trial, the pH of the MSA 7.5 and MSA 10 soils was 7.43 ± 0.04 and 7.76 ± 0.05, respectively, whereas for the MSA 5 soil the pH was 7.11 ± 0.03 (Figure S6A)."

Those lines refer to the treatment called MSA 1.5, where the application rate is equivalent to 30 tonnes ha^{-1}, the most similar to the application rate used in this study. Although there was no difference from the starting pH with that treatment, the difference compared to the control soil was ~0.3 pH units at the end of the study, which we should have been referring to.

We've modified that section to read:

The mean difference in soil pH seen here is in the same order of magnitude as that observed in a study using a rock containing the faster dissolving mineral, wollastonite (a mineral that dissolves up to 1200 times faster than faster weathering

silicate minerals [45]), applied at 30 tonnes ha^{-1} (mean difference of 0.3 pH units to the control soil after 98 days, [50]). The starting pH of the soil in [50] was similar to the circumneutral pH of the soil in this study. In more acidic soils even larger changes in soil pH have been observed, e.g. [58] reported a change of 0.5 pH units (from 4.7 to 5.2) within 9 months of application of 20 tonnes ha^{-1} basalt. Although, dissolution rates would be expected to be more favourable in the study by [58], where the particle size used was less than 250 µm, and the study was conducted in a Malaysian cocoa plantation with tropical temperatures and precipitation.

Line 337: but there is no evidence in this study of what these potassium-bearing basaltic phases are... If the liming effect is due to calcite, as suggested earlier, than how is a significant amount of K being released? Also back to the statistics, looking at Table 2 it is hard to believe that the K values are statistically different. In the grain, the values are 0.42 +/- 0.04 versus 0.44 +/- 0.04 for direct drill, which are very much overlapping values, and 0.39 +/- 0.03 versus 0.42 +/- 0.03 for ploughed, which also are perfectly overlapping values.

As shown through the mineral stoichiometry in Table 1 and referred to in line 386, K is thought to be present in the illite/mica phase. There could also be K in the amorphous phase, but as stated in lines 189-192, without SEM-EDS analysis we cannot determine the chemical composition of this phase.

Potassium release may have originated from the illite/mica phase identified through XRD, as potassium is a key component of illite and several mica minerals (e.g. muscovite and biotite).

The difference in the absolute mean +/- standard deviation of potassium concentrations does indeed appear modest, especially due to the large variability and hence standard deviation within each group. The statistical test employed, linear mixed effect modelling, is used because the effect of different treatments (fixed effects) can be assessed in datasets where several intended or unintended parameters affect the end result. As mentioned in another question regarding the statistics, we are also comforted with the fact that we were able to reproduce similar p-values using a two-way ANOVA including the block term.
Similarly to the yield response, we see an overall tendency towards higher uptake of potassium both in the tissue and grain samples:

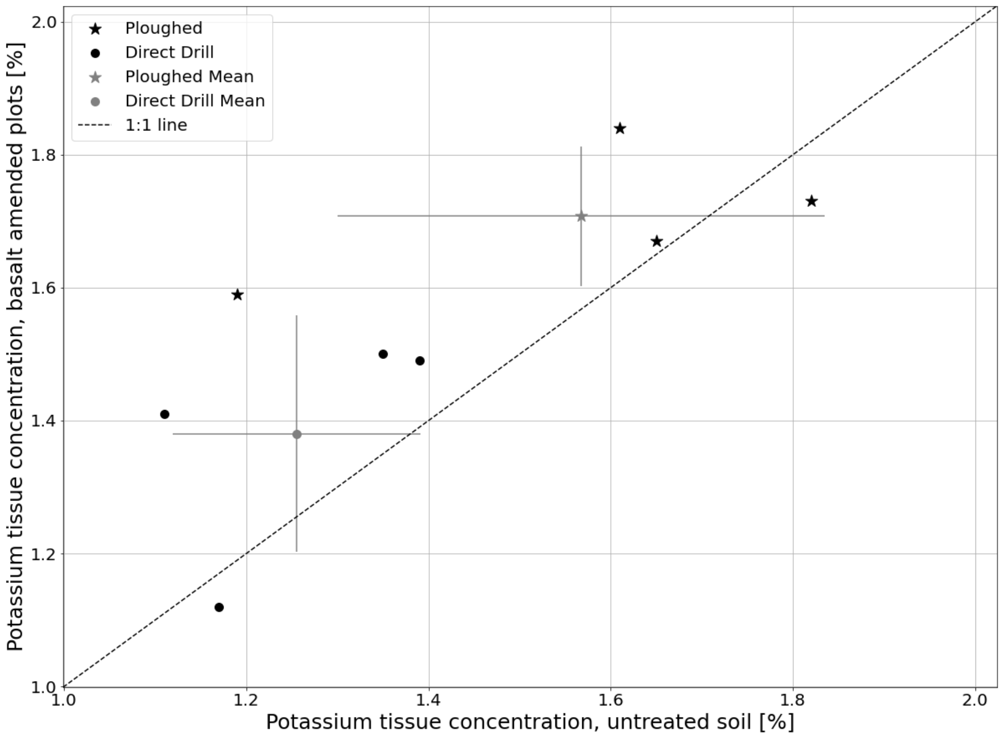

Ploughed
Direct Drill
Ploughed Mean
Direct Drill Mean
1:1 line
Potassium tissue concentration, basalt amended plots [%]
Potassium tissue concentration, untreated soil [%]
1.0
1.2
1.4
1.6
1.8
2.0

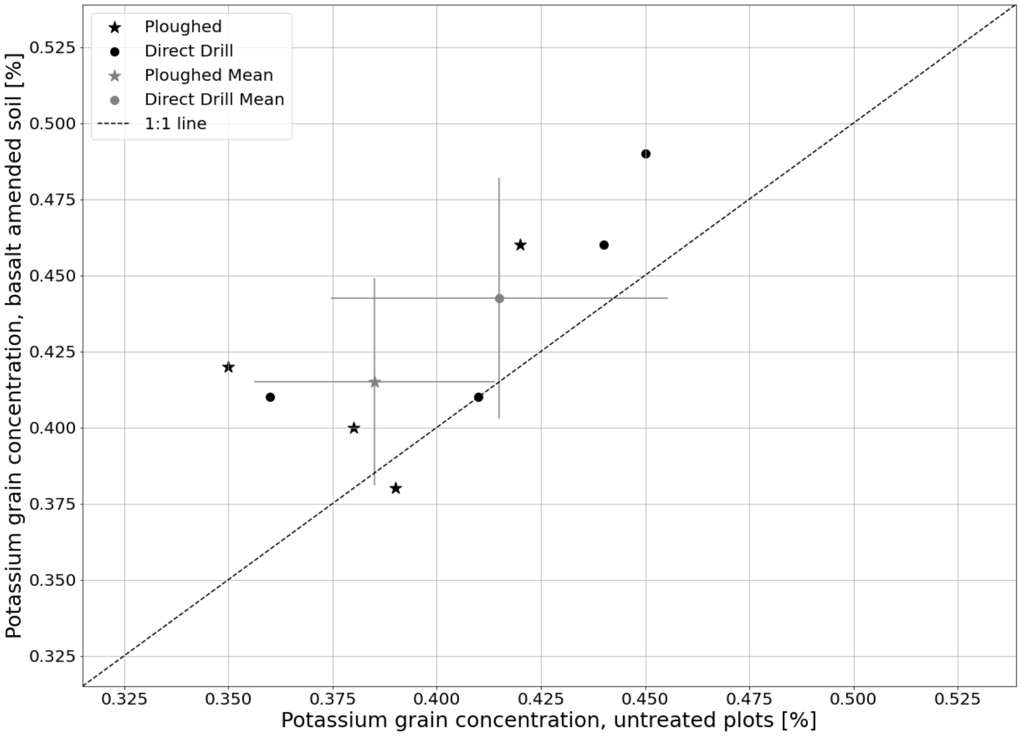

Lines 338 to 346 attempt to explain possible mechanisms, but very hypothetically. The odds that other soil effects are responsible for the small changes in K uptake by plants, such as cation exchange with innate K in soils, are likely larger than to attribute to the small odds that poorly weathered minerals incongruently released K in soils. If this was the case, soil remineralization would already have overtaken the use of potash in many countries that have a hard time affording to import potash.

We have added a comment that the K on soil exchangers could also be mobilised as a result of the release of other faster-weathering minerals replacing the innate K present on exchange sites. However, just because a mineral dissolves slowly, this does not mean that it will not dissolve and contribute to the pool of K in soil porewaters.

Regarding the part of the question that is 'why hasn't remineralisation taken over potash' - as you have mentioned before, the K increases are modest and are not likely to replace the K-fertiliser requirements. Simulations which have determined the rate of K-release from basalts, based on mineralogy, have found that basaltic materials typically do not supply enough K over a 5 year period to replace fertiliser application rates for arable crops in the UK (Lewis et al., 2021).

We have edited this paragraph to account for the changes suggested:

"The increase in tissue and grain potassium is not likely to have occurred as a result of the change in pH. The potassium may have originated from a variety of sources including: (1) the rock, (2) the soil and (3) via symbiotic plant-microbe relationships.

Considering the rock, potassium~~*potassium release*~~ *may have originated from the illite/mica phase identified through XRD, as potassium is a key component of illite and several mica minerals (e.g. muscovite and biotite).* ***This phase is generally considered to have a medium dissolution rate, but will still dissolve and may release additional potassium into the soil solution. Additionally,*** *similar to calcium and magnesium, potassium* ***within the illite/mica phase*** *may be preferentially released into the soil solution before the silicon-oxygen bonds are broken, as hypothesised [55] and observed [56] in work elsewhere.****The weathering of faster weathering minerals may have released elements (e.g. Ca and Mg) which could replace innate K from the exchange sites, making it more readily available for plant uptake. Additionally, g****iven the biotic nature of soils, the potassium may have also been preferentially mined from the mineral structure by microorganisms, including mycorrhizal fungi [57], though this hypothesis was not investigated in this study."*

General comment: it is commented in the text that basalt may have impacted draught resistance. One effect that mineral amendments can have is in improving soil texture, and hence improving water infiltration and retention near the plant roots. In another basalt study that I have reviewed, that seemed to largely explain an effect that was claimed. Perhaps this effect should be considered in hypothesizing reasons for the short-term effects seen.

This is an interesting point and we would have liked to include it in the section around the environmental impacts on the effect of basalt amendment in the yield discussion. Unfortunately, we were not able to track down the mentioned publication.

Conclusions: based on the aforementioned comments, some of the conclusion statements could be reconsidered. The conclusion does speak to the pH effect being a large effect, and does pose that calcite rather than silicate dissolution may explain much of the results. Still, there are comments about potassium that seems to be stretching the certainty of the results, and the last statement that "crops amended with crushed basalt are safe for consumption" is far too over-reaching considering how diverse basalt composition can be. This type of statement can be taken very much out of context in the MRV world. The conclusion section, which many reader read more than the rest of the text, should be more balanced to inform the reader than this was a short-term experiment under less than ideal field conditions and that many of the results are not well understood as there is no supporting mechanistic evidence provided in this study being the comparative analyses that are prone to variability effects and effects not considered in the study.

Thank you for the feedback on the conclusion. We've amended the conclusion to be more balanced, highlighting that the results are only from one growing season and that this growing season was particularly exceptional.

Reviewer #2: This manuscript has discussed the effect of applying basalt powder to the soil, leaf tissue and grains of oat plants in a temperate climate. This information is important for the academic community and especially for farmers. The manuscript is well written and its discussions are well reasoned. Authors can find some suggestions/annotations in the attached file. I recommend the approval of the manuscript if the due suggestions are duly answered or justified.

---

6. PLOS authors have the option to publish the peer review history of their article (what does this mean?). If published, this will include your full peer review and any attached files.

If you choose “no”, your identity will remain anonymous but your review may still be made public.

Do you want your identity to be public for this peer review? For information about this choice, including consent withdrawal, please see our Privacy Policy.

Reviewer #1: **Yes:** Rafael M. Santos

Reviewer #2: No

[NOTE: If reviewer comments were submitted as an attachment file, they will be attached to this email and accessible via the submission site. Please log into your account, locate the manuscript record, and check for the action link "View Attachments". If this link does not appear, there are no attachment files.]

While revising your submission, please upload your figure files to the Preflight Analysis and Conversion Engine (PACE) digital diagnostic tool, https://pacev2.apexcovantage.com/. PACE helps ensure that figures meet PLOS requirements. To use PACE, you must first register as a user. Registration is free. Then, login and navigate to the UPLOAD tab, where you will find detailed instructions on how to use the tool. If you encounter any issues or have any questions when using PACE, please email PLOS at figures@plos.org. Please note that Supporting Information files do not need this step.

5. Review Comments to the Author

Reviewer #2:

Line 80-83: Improve it!

Add Avena sativa to the crop field item.

Has been modified to emphasize that it’s an agronomic study, focusing on effects observed during the first growing season.

Line 102: Present the information in this topic in chronological order, to facilitate understanding.

Amended accordingly and ‘exclusively’ added to emphasise that basalt was not incorporated down to the plough depth in the ploughed fields, since ploughing was done prior to basalt application:
The ploughed fields were tilled to a depth of 15-30 cm at the end of March, followed by a press within three days (using Simba Unipress). Basalt was applied on Apr 12th 2022 using

a conventional agricultural lime spreader. The target application rate was set to 20 tonnes ha^{-1}, as this is the maximum the commercial spreader can do in a single pass, the actual mean spreading rate was determined to be 18.86 tonnes ha^{-1}. The ploughing was done prior to basalt application, hence the basalt was exclusively incorporated to the seeding depth across both cultivation types.

Line 105: Why did the authors choose this dose? Justify!

Text has been modified to explain why the spreading density landed on an average of 18.86 tonnes ha^{-1}, see amended text for previous question.

Line 136-141: Citation?

We were only able to obtain this information following email correspondence with Lancrop Laboratories. Citation has been included stating: Lancrop laboratories, personal communication.

Line 149-151: Citation?

We were only able to obtain this information following email correspondence with Lancrop Laboratories. Citation has been included stating: Lancrop laboratories, personal communication.

Line 169: Citation:

E.g. Shapiro and Wilk 1965

Thank you for making us aware of the lack of a reference.

Line 177: Seabold, Skipper, and Josef Perktold. “statsmodels: Econometric and statistical modeling with python.” Proceedings of the 9th Python in Science Conference. 2010.

Thank you for making us aware of the lack of a reference.

Table 2: In future works, pay more attention to the number of repetitions/block. For data such as productivity, it is known that large plots, as used in this work, are ideal. However, the researchers could have increased the number of blocks to have a larger value of n. This increases the degree of freedom of the ANOVA.

We agree with the reviewer that the study would have benefitted from a larger number of plot replicates. This trial (which was the first to be set up by UNDO in collaboration with Newcastle University) was conducted on existing plots that were established ~20 years ago, hence the number of plot replicates was decided upon apriori to the start of this project.

Line 214: I suggest the authors these articles for your discussion.

Luchese, A.V., de Castro Leite, I.J.G., Alves, M.L. et al. Can Basalt Rock Powder be Used as an Alternative Nutrient Source for Soybeans and Corn?. J Soil Sci Plant Nutr (2023). https://doi.org/10.1007/s42729-023-01322-3

Luchese A.V., Gutz de Castro Leite I.J., da Silva Giaretta A.P., Alves M.L., Pivetta L.A., Missio R.F. Use of quarry waste basalt rock powder as a soil remineralizer to grow soybean and maize (2023) Heliyon, 9 (3), e14050 DOI: 10.1016/j.heliyon.2023.e14050

Korchagin J., Escosteguy P.A.V., Bortoluzzi E.C. Nutrient transfer in rangelands under rock powder amendment (2022) Journal of Plant Nutrition and Soil Science, 185 (5), pp. 656 - 667 DOI: 10.1002/jpln.202200059

Thank you for the suggestions for extra references for the discussion. We've included the first two, but found it difficult to find a suitable spot for Korchagin et al., 2022, as that study relates to rangeland soil that has never received fertiliser and as such is a bit for from the more intensive agriculture this study revolves around.

Line 404-406: The authors sometimes mention "poor emergence", however they never present the stand of plants in the experiment. In my opinion this is important information for the manuscript.

Indeed, a measure of the emergence, count of number of ears per square metre or number of tillers would have been good measures to include, underlining the poor emergence of plants on the direct drill plots. Observations of the plots were made throughout the growing season and although two different cultivation methods were employed, the main focus of this study was to evaluate the effect of basalt amendment. The observations made on the emergence of the plants were on the direct drill plots compared to ploughed plots and were observed both on the control and basalt amended plots. As the observed poor establishment of oat plants did not appear to be related to the basalt amendment, further analysis of the stand of plants was not prioritised. The 'poor emergence' is primarily mentioned when discussing differences in yield and crop nutrients between the two cultivation types.
