# Supplementary material for: Initial agronomic benefits of enhanced weathering using basalt: A study of spring oat in a temperate climate
Source: PLoS One. 2024 Mar 27;19(3):e0295031. doi: 10.1371/journal.pone.0295031 (PMC10971544; doi:10.1371/journal.pone.0295031)
Supplement: Attachment — Submitted filename: ResponseToReviewers_II.pdf [file pone.0295031.s006.pdf]

Thank you very much for the comments on our manuscript. Please see responses in italic. We have also taken the opportunity to improve language and correct spelling mistakes throughout the manuscript, see manuscript with tracked changes.

PONE-D-23-15404R1
Initial agronomic benefits of enhanced weathering using basalt: A study of spring oat in a temperate climate
PLOS ONE

Dear Dr. Skov,

Thank you for submitting your manuscript to PLOS ONE. After careful consideration, we feel that it has merit but does not fully meet PLOS ONE's publication criteria as it currently stands. Therefore, we invite you to submit a revised version of the manuscript that addresses the points raised during the review process.

Please submit your revised manuscript by Nov 29 2023 11:59PM. If you will need more time than this to complete your revisions, please reply to this message or contact the journal office at plosone@plos.org. When you're ready to submit your revision, log on to https://www.editorialmanager.com/pone/ and select the 'Submissions Needing Revision' folder to locate your manuscript file.

Please include the following items when submitting your revised manuscript:

- A rebuttal letter that responds to each point raised by the academic editor and reviewer(s). You should upload this letter as a separate file labeled 'Response to Reviewers'.
- A marked-up copy of your manuscript that highlights changes made to the original version. You should upload this as a separate file labeled 'Revised Manuscript with Track Changes'.
- An unmarked version of your revised paper without tracked changes. You should upload this as a separate file labeled 'Manuscript'.

If you would like to make changes to your financial disclosure, please include your updated statement in your cover letter. Guidelines for resubmitting your figure files are available below the reviewer comments at the end of this letter.

If applicable, we recommend that you deposit your laboratory protocols in protocols.io to enhance the reproducibility of your results. Protocols.io assigns your protocol its own identifier (DOI) so that it can be cited independently in the future. For instructions see: https://journals.plos.org/plosone/s/submission-guidelines#loc-laboratory-protocols. Additionally, PLOS ONE offers an option for publishing peer-reviewed Lab Protocol articles, which describe protocols hosted on protocols.io. Read more information on sharing protocols at https://plos.org/protocols?utm_medium=editorial-email&utm_source=authorletters&utm_campaign=protocols.

We look forward to receiving your revised manuscript.

Kind regards,

Timothy Omara, PhD
Academic Editor
PLOS ONE

Journal Requirements:

Please review your reference list to ensure that it is complete and correct. If you have cited papers that have been retracted, please include the rationale for doing so in the manuscript text, or remove these references and replace them with relevant current references. Any changes to the reference list should be mentioned in the rebuttal letter that accompanies your revised manuscript. If you need to cite a retracted article, indicate the article's retracted status in the References list and also include a citation and full reference for the retraction notice.

All references have been checked, the reference list is complete and correct.

[Note: HTML markup is below. Please do not edit.]

Reviewers' comments:

Reviewer's Responses to Questions

Comments to the Author

1. If the authors have adequately addressed your comments raised in a previous round of review and you feel that this manuscript is now acceptable for publication, you may indicate that here to bypass the "Comments to the Author" section, enter your conflict of interest statement in the "Confidential to Editor" section, and submit your "Accept" recommendation.

Reviewer #1: (No Response)

Reviewer #2: All comments have been addressed

---

2. Is the manuscript technically sound, and do the data support the conclusions?

The manuscript must describe a technically sound piece of scientific research with data that supports the conclusions. Experiments must have been conducted rigorously, with appropriate controls, replication, and sample sizes. The conclusions must be drawn appropriately based on the data presented.

Reviewer #1: Partly

Reviewer #2: Yes

---

3. Has the statistical analysis been performed appropriately and rigorously?

Reviewer #1: Yes

Reviewer #2: Yes

---

4. Have the authors made all data underlying the findings in their manuscript fully available?

The PLOS Data policy requires authors to make all data underlying the findings described in their manuscript fully available without restriction, with rare exception (please refer to the Data Availability Statement in the manuscript PDF file). The data should be provided as part of the manuscript or its supporting information, or deposited to a public repository. For example, in addition to summary statistics, the data points behind means, medians and variance measures should be available. If there are restrictions on publicly sharing data—e.g. participant privacy or use of data from a third party—those must be specified.

Reviewer #1: Yes

Reviewer #2: Yes

---

5. Is the manuscript presented in an intelligible fashion and written in standard English?

PLOS ONE does not copyedit accepted manuscripts, so the language in submitted articles must be clear, correct, and unambiguous. Any typographical or grammatical errors should be corrected at revision, so please note any specific errors here.

Reviewer #1: Yes

Reviewer #2: Yes

---

6. Review Comments to the Author

Please use the space provided to explain your answers to the questions above. You may also include additional comments for the author, including concerns about dual publication, research ethics, or publication ethics. (Please upload your review as an attachment if it exceeds 20,000 characters)

Reviewer #1: The authors have made useful revisions, though there still a couple of topics to improve:

A) Soil pH results:

1) The manuscript says: "The mean difference in soil pH seen here is in the same order of magnitude as that observed in a study using a rock containing the faster dissolving mineral,

wollastonite (a mineral that dissolves up to 1200 times faster than faster weathering silicate minerals [45]), applied at 30 tonnes ha−1 (mean difference of 0.3 pH units to the control soil after 98 days [50]). The starting pH of the soil in [50] was similar to the circumneutral pH of the soil in this study."

The study [50] used soybean and alfalfa, which are both nitrogen-fixing plants, which therefore have an acidifying effect during plant growth. In the present study of the authors, the plant used was oat, which is not a nitrogen-fixing plant, and therefore does not cause soil acidification to the same extent. So a pH elevation in a soil where the plant is countering the liming effect is much harder to achieve. This can explain why a similar amendment rate of the much faster weathering silicate could have had a similar pH change effect to basalt. So the direct comparison should be done more caustiously.

That is a very good point and the manuscript has been amended accordingly. Thank you for drawing our attention to this aspect.

2) The manuscript says: "The significant first year differences in pH may be ascribed to the 3.6 wt.% content of faster dissolving calcite (Table 1). The calcite (calcium carbonate) fraction contribution in this study was 0.68 tonnes ha−1. Comparatively, if a limestone product with a 70% effective calcium carbonate equivalent and an application density of 2 to 5 tonnes ha−1 (similar to a limestone product used in [59] had been used, the contribution would range between 1.4 to 3.5 tonnes ha−1 calcium carbonate (between two and fivefold higher concentrations than that of the basalt). A study of six basalts from widely different geological provinces found that basalts contain up to 1.25 wt.% carbonates [60], compared to which the content in Divet Hill is relatively high."

The study [59] was comparing limestone applications between 1 to 4 tonnes/hectare to basalt application of 33 to 99 tonnes per hectare. Since the authors' present study used 18 tonnes/hectare, it is on the low end of the study [59]. In order to compare the present study to [59], rather than generaly comparing pH changes, one should use the empirical equations shown in Figure 1 of [59]. Those empirical equations show two things. One is that for limestone application, the pH effect (which is the slope of the fitted lines) is in the order of 0.148 to 0.269 pH units per tonne of limestone. Correting this for the liming efficiency reported in [59] (72.5%), we get 0.204 to 0.371 pH units per tonne of CaCO3. So if we take the 0.68 tonne/hectare value stated in the present study as the rate of CaCO3 addition to the soil, we would expect to see 0.14 to 0.25 pH units increase just due to the CaCO3 amendment effect, which in in line with the observed pH changes. So study [59] helps to confirm that most of the pH effect observed is likely to be attributable to the CaCO3 content of the basalt. Notably, in study [59], they report that the effect of basalt amendment on pH was only 0.00022 pH units per tonne of basalt amendment (for one soil, for the other the effect was negative, so basalt did not prevent the soil from becoming naturally more acidic). It may be that the basalt used in [59] contained less CaCO3.

The intention of the above section was to compare the amount of calcite contributed through the Divet Hill basalt with a typical lime application rate for the region where this study was conducted (2-5 tonnes ha^{-1}). Admittedly, that did not come across very clearly, especially not when assessing how the reviewer interpreted that paragraph. However, the intention in this paragraph wasn't to compare results from this study to the pH changes reported in that

paper. The paragraph has been modified to underline the point that the particular basalt used in this study (Divet Hill basalt), applied at a rate of 18.86 tonnes ha^{-1}, does not contain enough calcite to fully substitute for a typical single lime application.

It's an interesting exercise to use the empirical slope (change in pH unit per tonne lime) from Figure 1 in [59] to estimate what the pH change in this study would have been, had all environmental conditions been equal and assuming all of the basalt-derived calcite had dissolved. However, as presented in Figure 1 of [59], the slope may be markedly different when considering another crop type (the gradient used in the above example relates to maize, but the gradient for soy is steeper). Furthermore, as the variability in those empirical regressions indicate, the effect of one tonne of calcium carbonate on the change in soil pH is affected by the buffering capacity of the native soil. Other aspects influencing the soil pH include (but are not limited to) the decomposition of organic matter and different strategies for incorporating amendments into the soil.
Regarding the referenced slope value for basalt amendment, we are unable to find the number '0.00022' in Figure 1 in [59]. The slope relating to basalt amendment of a sandy clay loam soil in [59] (which appears to be a similar texture to the soil used in this study - see line 109, page 4) with maize (a different crop to the oat used in this study) is an order of magnitude higher, 0.0073 pH units per tonnes of basalt per hectare. Applying this slope to an application rate of 18 tonnes ha^{-1}, the predicted increase in pH would be around 0.13 units. Whether the basalt used in [59] contained less calcite than the Divet Hill basalt is unknown, as the carbonate composition was not reported in [59].
Accordingly, we are reluctant to apply the empirical regressions from [59] to the soil and crop type used in this study, as they may relate to incomparable experimental conditions.

We do not discount the effect that the calcite content in the Divet Hill basalt has had on soil pH; indeed, most of that paragraph is alluding to the importance of the calcite content in the basalt (which we hope has now become clearer, lines 349-364). But we maintain that the relative contribution of the mineral reactions to the change in soil pH cannot be determined with the present dataset. One reason for this is that there are no data on how large a proportion of the 3.6 wt.% of calcite actually dissolved. The dissolution rate of calcite is very fast, but some portion of the calcite may remain undissolved inside the basaltic grains, given that the particle size ranges up to 4 mm.

This bring the second point, which is that the authors state that most basalts have no more than 1.25 wt% $CaCO_3$, while the one used in the present study had three times this value.

To clarify, we state that a study [60] examining six different basalts found that the maximum carbonate content was 1.25 wt.%, and posit that feedstocks with relatively high calcite proportions may be of interest to farmers, if they are looking to see pH-related effects within the first year. We do not make reference to the average carbonate concentration of basaltic rock

We have changed the wording to make the above point clearer (lines 359-360), from:

A study of six basalts from widely different geological provinces found that basalts contain up to 1.25 wt.% carbonates [60]

To:
A study of six basalts from different geological provinces identified carbonate concentrations up to 1.25 wt.% [60].

So more reasoning to attribute the pH effect to CaCO3 content for the duration of the present study. The manuscript already states this, deep in the discussion, but not as clearly or quantitatively as I just described. Most notably, in the abstract, it is simply stated that the pH effect observed is "likely due to rapidly dissolving minerals". This is very vague, it suggest that basalt has rapidly reactive silicates that the study found conclusively to contribute to this effect, but that is not the case. The abstract needs to be more precise on this point since it is what most readers will read.

We are grateful for the reviewer's feedback on this point. Yes, we agree that the abstract should have been more explicit in stating that the first-year effect on the soil pH is likely to be heavily influenced by the calcite content of this particular basalt. Calcite is now explicitly mentioned in that sentence in the abstract, and is listed as the likely primary factor controlling soil pH in the conclusion.

B) EW data:

Considering that the study was done in close cooperation with an MRV company, and several authors are associated with the MRV company, it is odd that no MRV data is available to be reported in this study. As the authors state, the uptake of potassium by the plants and its accumulation in grains could be a sign of weathering, but that assumes that the element is present only in the identified silicate phases, and not as a readily dissolvable salt in the amorphous or unidentified phase. Plant availability testing (i.e., leaching tests) should be done to confirm if the release of K can really be attributed to EW or simply to the amendment or to other soil changes. Therefore, if the K uptake, is not a true sign of EW, and the soil pH change is largely due to CaCO3, is this really an EW study, or is it called an EW study because of the involvement of an MRV company?

Lets imagine that it was a different academic research group that did this experiment, without involvement of an MRV company. Either (a) they would not use the term "enhanced weathering" to describe their research (as seen in many papers that have tested basalt as a soil remineralizer or liming agent), or (b) they would have looked for more evidence of EW happening by analizing soils and porewater.

So the situation of this manuscript is overall odd in terms of how it is advancing the science, and not just how it will be a useful paper for an MRV company to refer to when saying that the technology is scientifically sound. I am especially saying this because I know that the academic group attached to this work is capable of publishing very scientifically interesting and rich studies.

Following the points raised above (section 6.B) and during the last round of review on the K uptake, we have further revised the discussion (see manuscript changes in the 'Nutrient uptake' chapter).

In the above feedback, several other points are raised. We have split those into the following sections:

No MRV data

The present study is an agronomy trial, which did not seek to quantify rock weathering or carbon dioxide removal (CDR) rates. We chose the title for this paper such that it would accurately reflect the data being reported, namely, the agronomic effects related to the enhanced weathering of basalt added to agricultural soil under oat cultivation.

Amorphous phase of the basalt

The available primary data are insufficient to either support or dismiss the reviewer's suggestion that the amorphous phase contains a readily-dissolvable K-bearing salt(s).

Source of K and leach testing

As listed in the discussion of the significantly higher tissue and grain K uptake, there are several possible sources for this nutrient:, 1) the rock, 2) the soil and 3) plant-microbe interactions. We posit that some of the K may have come from silicate weathering, but the discussion does not rule out other K sources, and several possibilities are mentioned.

It may be that the significantly higher uptake of K is partly due to K being more available to plants grown in the basalt-amended plots, due to native soil K being released from the soil cation exchange reservoir through competitive adsorption with other (potentially, rock-derived) cations. In this case, the effect is also attributable to the basalt amendment.

A leach test of the basalt feedstock could give an indication of whether the basalt contributed any readily-available K, and hence, help to reveal the mechanisms of K uptake. But the relative contribution from any readily-available K versus other potential sources would be difficult to establish with a leach test alone. This study was not designed to identify the precise mechanism(s) for K-fertilisation, but ultimately, the evidence suggests that using the Divet Hill basalt amendment may increase grain K, which has agronomic value, regardless of the precise mechanism for K delivery. The different possible sources of K are discussed in lines 392-407.

We thank the reviewer for their comments on the potassium aspect of the discussion, and have revised this section to make the intended points clearer (Lines 390-391; 401-404)

Not an EW study

The reviewer has suggested that our study is not an EW study, primarily because: 1) there is no quantification of the CDR potential, and 2) because the change in soil pH may be ascribed the calcite dissolution and 3) because K may originate from other sources than silicate weathering (responded to in the above section).

1) This study assessed the agronomic implications of using basalt as an EW amendment - an important aspect of this promising CDR strategy, where deployment on agricultural fields (with minimal land-use conflicts) is viewed as a key advantage. To investigate the safety and potential for detrimental effects on crop yield, it is essential to assess EW using different

feedstocks, soil types, crop varieties, and climate regions. This study is one such assessment, and to our knowledge, the first in this geographical region using basalt at an application rate of around 20 tonnes ha^{-1} with this soil type and crop.

Positioning an agronomy trial such as ours as an EW study is not unprecedented in the literature; indeed, we reference another agronomy-focused EW study [22] in our manuscript. In this study the EW feedstock is Greenlandic glacial rock flour, 'enhanced mineral weathering' is mentioned in the first sentence in the abstract and 'enhanced rock weathering' and 'ERW' are stated as keywords. This is despite the results reported being entirely agronomic, with no estimation of rock weathering or CRD rates, and the research group being ostensibly unconnected to a commercial EW company.

While research in estimating and quantifying the amount of carbon dioxide sequestered following basalt application is also a very important aspect of EW, we do not agree that experiments focusing on agronomic effects should be barred from classification as EW studies. Indeed, we believe such studies are necessary to demonstrate the scalability of the technology and to better quantify the co-benefits for landowners.

2) While silicate minerals have the greatest long-term potential in terms of carbon dioxide removal (CDR), we would further argue that dissolution of carbonate minerals in EW feedstocks is also enhanced weathering. Recent publications (that are ostensibly EW papers) lend support to this viewpoint, e.g. 'The efficacy of enhancing carbonate weathering for carbon dioxide sequestration' by Knapp and Tipper (Front. Clim., 11 August 2022, Sec. Carbon Dioxide Removal, Volume 4 - 2022, https://doi.org/10.3389/fclim.2022.928215). In their publication 'soil dissolution' of carbonate minerals is highlighted as a rapid pathway to enhanced weathering, and the keywords list includes 'enhanced weathering in soil'.

Soil and porewater samples

The reviewer correctly identified that this study was a collaboration between UNDO Carbon Ltd and Newcastle University, conducted at the Newcastle University-owned research farm in Nafferton. The objective of this study was to investigate agronomic effects related to the use of crushed basalt rock for EW purposes on agricultural land, especially the effect on crop yield and plant uptake.

While we agree with the reviewer that regular sampling of soil and porewater throughout the growing season would have added value to the study, the primary aim was to assess the agronomic effects. As discussed during the first round of revisions, it may well be that the first signs of weathering will be found in the differences in yield and not necessarily on e.g. the soil cation exchange pool, especially when operating under field conditions, with crushed basalt rock, particle sizes up to 4 mm and an application rate of around 20 tonnes ha^{-1}.

'MRV' company

We believe that the findings in this study should be evaluated on the basis of scientific merit, regardless of any involvement with different companies, people or research groups.

Moreover, addressing the reviewer's comments on this matter, we would like to clarify that UNDO Carbon Ltd is an EW company, not an MRV company, and is actively deploying

crushed basalt rock to agricultural land in NE England and Scotland. As such, UNDO is interested in all aspects of EW, including any potentially negative effects of the deployment of EW feedstocks. While UNDO is interested in collaborating with research groups to develop MRV approaches, the company is also driven to engage in research collaborations on other aspects of EW.

Scientific relevance of this study

We maintain that this study has scientific merit given that it is the first field-scale study using crushed basalt rock as an EW amendment to agricultural soil in NE England. To the best of our knowledge, it is also the first study with spring oats and the application rate was lower than any previous studies. Notably, the application rate in this study was determined by the maximum delivery capacity of a commercial spreader in a single pass, i.e. it demonstrated the use of existing agricultural infrastructure.
We believe that EW as a CDR technology will benefit from many more agronomic studies across different soil and crop types in temperate, as well as other climatic regions.

Reviewer #2: (No Response)

---

7. PLOS authors have the option to publish the peer review history of their article (what does this mean?). If published, this will include your full peer review and any attached files.

If you choose “no”, your identity will remain anonymous but your review may still be made public.

Do you want your identity to be public for this peer review? For information about this choice, including consent withdrawal, please see our Privacy Policy.

Reviewer #1: No

Reviewer #2: No
